# Template-based mapping of dynamic motifs in tissue morphogenesis

**DOI:** 10.1371/journal.pcbi.1008049

**Published:** 2020-08-21

**Authors:** Tomer Stern, Stanislav Y. Shvartsman, Eric F. Wieschaus

**Affiliations:** 1 Department of Molecular Biology, Princeton University, Princeton, New Jersey, United States of America; 2 Department of Chemical and Biological Engineering, Princeton University, Princeton, New Jersey, United States of America; 3 The Lewis-Sigler Institute for Integrative Genomics, Princeton University, Princeton, New Jersey, United States of America; 4 Center for Computational Biology, Flatiron Institute - Simons Foundation, New York, United States of America; Purdue University, UNITED STATES

## Abstract

Tissue morphogenesis relies on repeated use of dynamic behaviors at the levels of intracellular structures, individual cells, and cell groups. Rapidly accumulating live imaging datasets make it increasingly important to formalize and automate the task of mapping recurrent dynamic behaviors (motifs), as it is done in speech recognition and other data mining applications. Here, we present a “template-based search” approach for accurate mapping of sub- to multi-cellular morphogenetic motifs using a time series data mining framework. We formulated the task of motif mapping as a subsequence matching problem and solved it using dynamic time warping, while relying on high throughput graph-theoretic algorithms for efficient exploration of the search space. This formulation allows our algorithm to accurately identify the complete duration of each instance and automatically label different stages throughout its progress, such as cell cycle phases during cell division. To illustrate our approach, we mapped cell intercalations during germband extension in the early *Drosophila* embryo. Our framework enabled statistical analysis of intercalary cell behaviors in wild-type and mutant embryos, comparison of temporal dynamics in contracting and growing junctions in different genotypes, and the identification of a novel mode of iterative cell intercalation. Our formulation of tissue morphogenesis using time series opens new avenues for systematic decomposition of tissue morphogenesis.

## Author summary

Much like many ballet dances that are choreographed by combining the same movements in different ways, tissue morphogenesis relies on combinations of highly conserved sub- to multi-cellular behaviors, or motifs, such as cell division, death and intercalation. Algorithms for systematic mapping of morphogenetic motifs are essential for making the most of rapidly accumulating live imaging datasets. Here, we present a template-based strategy for motif mapping using an integrated time series data mining and graph theoretic approach. Rather than relying on a set of manually-crafted criteria, our algorithm identifies instances of the motif based on similarity to a user-provided example or template. This strategy allows our algorithm to accurately identify the complete duration of each instance and automatically label different stages throughout its progress, such as cell cycle phases during cell division. As an illustration, we mapped intercalary cell behaviors during convergence-extension movements in wild-type and mutant *Drosophila* embryos. Our results revealed statistical properties in the dynamics of T1-cell rearrangements and multicellular rosettes and identified a new mode of iterative cell intercalation. Our time series approach opens new avenues for systematic decomposition and analysis of tissue morphogenesis.

## Introduction

The formation of functional structures during animal development is governed by repeated use of highly conserved morphogenetic behaviors, such as convergence-extension movements and out-of-plane epithelial deformations [1–4]. These morphogenetic “subroutines” have common evolutionary origins and common dependences on cell- and tissue-level dynamics. For example, several aspects of mesoderm invagination in *Drosophila* embryos bear striking resemblance to optic cup formation in vertebrates, and both of these processes depend on spatiotemporal modulation of apical cell constriction [5,6]. Another example of a highly conserved morphogenetic behavior is provided by *Drosophila* germband extension, which results in an in-plane reshaping of a patterned epithelial sheet [7]. This convergence-extension process is orchestrated by contractile actomyosin cables that induce directed cell rearrangements, similar to what was observed during vertebrate neurulation [8]. Other morphogenetic events, such as the formation of tubular structures of trachea and salivary glands, involve both apical cell constrictions and directed cell rearrangements, relying on combinations of these dynamic building blocks [8–11]. Thus, morphogenetic processes appear to be assembled from commonly used morphogenetic motifs, including apical cell constrictions, cell intercalations, delaminations, collective divisions, and migrations [12–17].

While simple inspection of development has allowed us to discover and characterize these motifs and continues to reveal new ones, the large size and complexity of imaging datasets, with many cells, each of which can over time participate in multiple motifs, makes it essential to establish robust approaches to automated mapping of known morphogenetic behaviors. Previous searches for such “motifs” have generally imposed a set of manually engineered criteria, or “if-then” statements on the graph of cell connectivity [18–25]. Notwithstanding the significant contribution of these works, approaches based on manually crafted criteria frequently struggle with capturing motifs that manifest high temporal or geometric variability. In addition, most existing algorithms have been designed to identify motifs based on a single critical time point during the progression through the motif, such as the time of completing cytokinesis in a dividing cell. This strategy therefore becomes less suitable for analyzing temporal progressions. Here, we present a “template-based search” algorithm for tissue motifs in live imaging data. Similar to mapping a target motif within a sequence database using the BLAST algorithm, we pose the problem of finding matches to a target morphogenetic motif as a time series subsequence matching task [26,27]. As a first step, we transform a user-provided example of the target motif into a time series, forming a template that can be used in scanning a live imaging dataset. This template is then matched to all possible candidates within the dataset using dynamic time warping (DTW), which neutralizes the effect of variations in the temporal dynamics. In addition to identifying the cells involved, the matching to the template also accurately identifies the first and last time points of each detected event. Moreover, it allows for automated labeling of different periods throughout the progression of the motif, such as different cell cycle phases during cell division.

In addition to the use of templates, our strategy uses high-performance graph theoretic algorithms and pruning heuristics to cope with the high combinatorial complexity of searching dynamic subgraphs within large datasets. Once matches are identified, they are sorted based on their similarity to the template and assigned values that can be used in selecting data for further analyses. Quality validation against simulated data and a comparison to an existing algorithm confirms the high accuracy of our approach.

Our application of this strategy to live imaging data from wild-type and mutant *Drosophila* embryos demonstrates the potential of a time series framework and opens new avenues for systematic decomposition of morphogenetic processes.

## Results

### Motif mapping as a subsequence matching task

**Fig 1:**
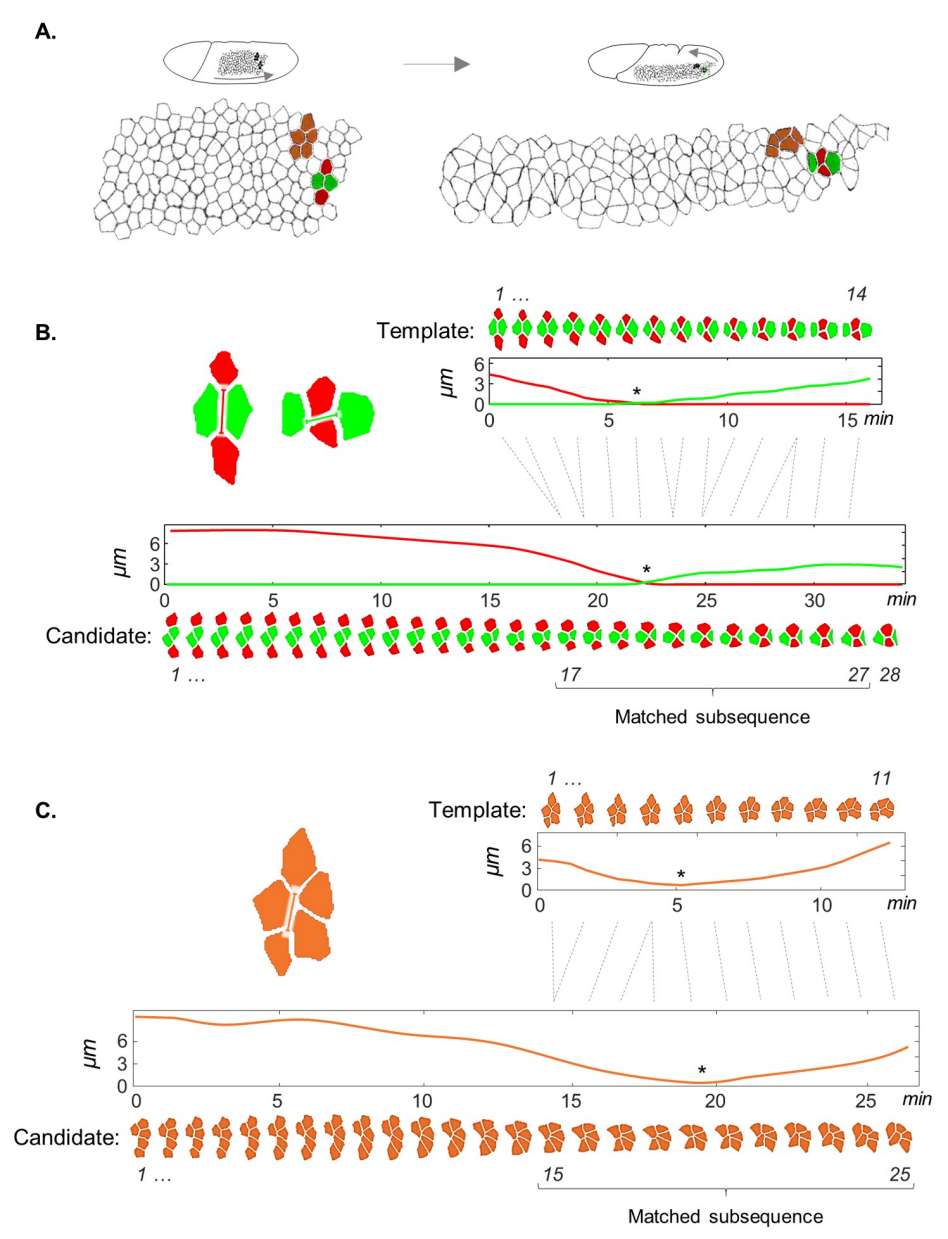
Motif mapping as a subsequence matching task. (A) Images of the early and late stages of *Drosophila* GBE, a convergence-extension movement that involves repeated cell intercalation events in the form of T1-transitions (red and green) or rosettes (brown). (B,C) Illustrations of optimal matchings (dashed gray lines) between all time points of the templates (top) and subsequences of time points within the candidates (bottom) in a T1-transition (B) and a 5-cell rosette (C). In (B) red and green curves represent the boundary distances between the two nearing (dorso-ventral) and the two parting (antero-posterior) cells, respectively. The transition time points are indicated by asterisks.

Given a user-provided example of a morphogenetic motif–a template–our algorithm identifies high confidence matches to this template in a live imaging dataset. To make things concrete, the main steps of the algorithm are outlined using data from live imaging of the fast phase of the GBE during *Drosophila* embryogenesis, a convergence and extension process during which the ventrolateral domain of the embryo nearly doubles its length while halving its height within ~30 minutes (Fig 1A). This extensively studied morphogenetic event relies on two types of intercalary cell behaviors: T1-transitions and multicellular rosettes. A T1-transition involves a group of four cells, which undergo a characteristic exchange of cell neighbors [28]. Rosettes involve five or more cells that converge to contact each other at a single vertex before resolving in the orthogonal direction [29]. Both of these motifs depend on contractile actomyosin cables, which in turn depend on the anterior-posterior (AP) patterning of the embryo [30]. Using live imaging datasets of membrane-labeled tissues we generated for this study, we demonstrate how our algorithm identifies high confidence matches to T1-transitions and rosettes and enables statistical analysis of their dynamics in wild-type and mutant embryos that lack AP patterning information.

In the first step of our approach, a representative example of a motif is converted into time series of features that provide a compact description of its dynamics. For a T1-transition, we used the minimal Euclidean distances connecting the six cell pairs in each four cell cluster (Fig 1B). Rosettes can be represented using just a single feature: the maximal pairwise distance between all pairs of cell boundaries at each time point (Fig 1C). From this point on, a motif template is a time series: $T=(\vec{t_{1}},\vec{t_{2}},\ldots ,\vec{t_{m}})$, where *m* is the number of frames included in the template and $\vec{t_{i}}$ is the vector of features describing the motif at the *i*^*th*^ time point. The dimension of this vector is equal to 6 for T1-transitions and 1 for rosettes. Next, the same features are evaluated for each candidate group of cells from the dataset that has the same number of cells as the target motif, and is tracked for *n* frames: $C=(\vec{c_{1}},\vec{c_{2}},\ldots ,\vec{c_{n}})$. Selection of candidate groups of cells is discussed in the next section. With this time series representation, our goal is to identify a subsequence *C*_*i*..*j*_ for 1≤*i*≤*j*≤*n*, that is similar to *T*.

Subsequence matching can be accomplished by “sliding” *T* over *C* and calculating for each matched subsequence the sum of Euclidean distances (EDs) between paired time points. Subsequences with small distances (low dissimilarity scores) indicate a match to the target motif and therefore detection of the event within the dataset. This approach assumes that the underlying processes advance at similar rates and can potentially overlook cell groups that advance slower or faster than the template. Instead, we perform subsequence matching using the Dynamic Time Warping (DTW) algorithm [31,32]. Unlike simple template sliding, DTW allows penalized stretching of sequences by repeating time points to optimize the match (Fig 1B and 1C). These stretching are similar in essence to gap openings in molecular sequence alignment. For instance, in Fig 1B the entire sequence *T* is warped against the subsequence *C*_17..27_:

$$WP^{T}:\;[1,\;2,\;3,\;4\;,\;5,\;6,\;7,\;8,\;9,\;10,\;11,\;12,\;12,\;13,\;14]$$

$$WP^{C}:\;[17,\;17,18,\;18,\;19,\;20,\;21,\;21,\;22,\;22,\;23,\;24,\;25,\;26,\;27],$$

where *WP*^*T*^ and *WP*^*C*^ are the warping paths of the template and the candidate, respectively. Time points in the candidate that are matched against multiple time points in the template indicate that the candidate advanced faster than the template, and vice versa. Theoretically, a single time point from one sequence can even be stretched to match the entire other sequence from start to end, thus resulting in unrealistic correspondence between the sequences. Such stretching can be avoided by constraining the optimization process, e.g. by setting an upper bound for the number of repetitions of a single time point, or an upper-bound for the slope of the warping path ([31,33]). Notably, DTW’s ability to overcome differences in the rates of the sequences makes it equally robust when the temporal sampling rate of the analyzed time-lapse is significantly different than this of the template.

For each detected subsequence, DTW calculates a score of its dissimilarity from the template, which is used to assess the likelihood of the subsequence to represent a true instance of the motif. The most commonly used score in DTW is the sum of EDs between vectors of features at matched time points:

$$DTW(T,C_{i..j})=\sum_{i=1}^{\left|WP^{T}\right|}\left\|\vec{T(WP_{i}^{T})}-\vec{C(WP_{i}^{C})}\right\|,$$

where |*WP*^*T*^| = |*WP*^*C*^| is the length of the warping path and ‖*x*‖ is the Euclidean norm of *x*. Since longer warping paths lead to summing of more terms and thus increase the evaluated dissimilarity, this scoring method might favor a shorter match over a longer one despite the candidate may be less similar to the template. To correct for this bias, we normalize the obtained score by the length of the warping path: $\hat{DTW}(T,C_{i..j})=DTW(T,C_{i..j})/|wp^{T}|$, which can be interpreted as the average ED between matched time points. Taken together, our subsequence matching approach provides a flexible way for detecting similarities to a template motif in live imaging data.

### Generalization to whole tissue motif mapping

So far, we have explained how a motif template is matched to a single candidate sequence. To do this for the entire imaging dataset we must first extract all candidates for such matching. In our case, we need all cell quartets for T1-transitions and all groups of 5 or 6 cells for cell rosettes, along with the time intervals during which the candidate sequences are present in the dataset. Cells within these groups must form a connected component (CC), which means that each cell can be reached from any other cell by a path within the component itself.

In graph theoretic terms, the task of identifying all CCs of a specific size is known as generating all connected induced subgraphs of size *k* (i.e. groups of *k* connected cells), which we apply to the adjacency graph of all cells in a single frame [34]. For large *k* values, generating all CCs is a demanding computational task. However, since the number of neighbors of a cell is typically less than ten, the graphs describing cell connectivity in these tissues are very sparse. This makes our problem ideally suited for ConSubg, a high-performance algorithm for this purpose. ConSubg exploits properties in the structure of the graph to avoid traversing through groups of disconnected vertices, and is particularly advantageous for large sparse graphs [35,36]. As an example, a snapshot of the germband midway through extension has 373 cells, 14,156 cell quartets, and 58,858 and 253,991 groups of 5 and 6 cells, respectively. On an average computer, these connected cell groups are identified within seconds by ConSubg. We tracked each of these groups over time, forming a list of candidates that are ready for template matching.

Like in a BLAST search, the matched subsequences are returned sorted from most to least similar. Depending on the subjective interpretation of the user, not all matches are similar enough to be considered true detections. One way to filter out false matches is by manually setting a cutoff score for the dissimilarity to the template. However, this approach works only when the target motif has a highly distinctive phenotype, such as the sequence of cell division phases. Otherwise, any selected threshold would result either in high false positive or high false negative detection rates, which is what we found for T1-transitions and rosettes. As an alternative, we implemented a supervised learning classifier based on the 1-Nearest Neighbor (1NN) rule. Using a graphical interface, the user is presented with the sorted list of candidates and asked to label a representative collection as ‘true’ or ‘false’ detections, thereby establishing a training-set. Then, each of the remaining (unlabeled) candidates is automatically labeled according to the most similar (1NN) candidate from within the training set. The similarity between the unlabeled and the labeled sequences is defined as the score of their DTW alignment. The results of this classification process are then shown to the user, which can iteratively improve the classification by relabeling candidates until satisfying accuracy is achieved. Notably, the simple 1NN classifier together with the DTW dissimilarity score still belongs to the state of the art, and is reported to be exceptionally difficult to beat [37,38] (see S1 File).

### Search space pruning

**Fig 2:**
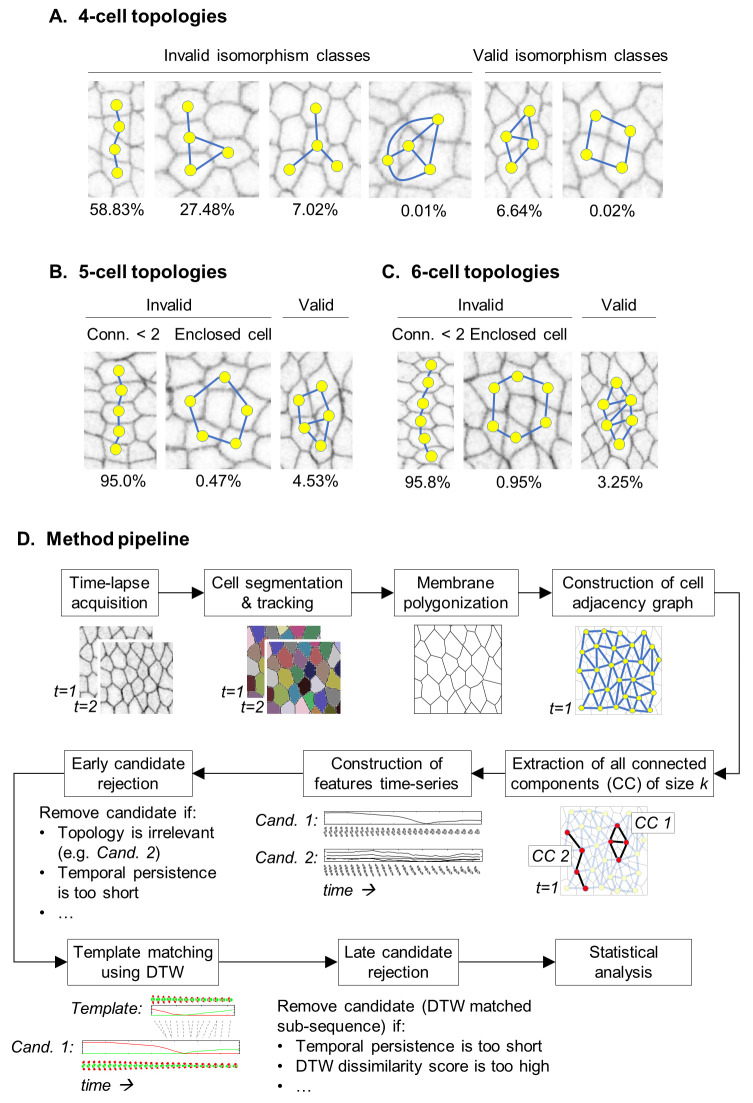
Early candidate rejection using a graph theoretic approach and method pipeline Examples of 4, 5, and 6 cell configurations and their empirical frequencies in imaging data. (A) All possible 4-cell configurations. The two configurations relevant for T1-transitions account for only 6.66% of the dataset. (B) Examples of 5-cell configurations. Valid configurations account for 4.53% of observed 5-cell connected components. (C) Valid and invalid 6-cell configurations. (D) Summary of the method’s pipeline demonstrated on the search of T1-transitions.

The number of candidate sequences with correct number of cells is very large, even for modestly sized datasets. However, fortunately, most of them can be rejected before moving on the computationally expensive steps of subsequence matching and scoring. To this end, we implemented a set of early rejection criteria. These criteria limit the search space by pointing to sequences or subsequences that are irrelevant for the target motif and remove them efficiently from the dataset. Some of these criteria come from considering the adjacency relations of cells in connected components. In particular, since only two out of six possible cell arrangements for cell quartets are observed in T1-transitions, all other alternatives can be excluded from consideration (Fig 2A). Similarly, when considering rosettes, we require that each cell within a connected component has at least two neighbors (Fig 2B and 2C). We also exclude components that contain fully surrounded cells that do not belong to the component (Fig 2B and 2C).

All of these criteria can be formulated in compact graph theoretical terms. For instance, testing whether a given cell quartet has the right topology, amounts to evaluating planar graph isomorphisms, a well-defined problem with efficient algorithmic solutions [39]. Testing whether a connected component contains fully enclosed cells can be done using the Euler characteristic formula for planar graphs. In addition to excluding cell groups with irrelevant connectivity patterns, we exclude sequences that are not sufficiently persistent (e.g., exist less than 2.5 minutes) and those that do not include an event of neighbor exchange. Taken together, these criteria reduce the volume of search space by 98%. Notably, the same strategy can also be used after subsequence matching to reduce the number of candidates to be classified. Since our analysis of live data will be centered on the temporal dynamics of motifs, we used these criteria to filter out intercalary events that persist for less than 2.5 minutes. In addition, we filtered out T1-transitions in which the initial length of the contracting junction or the final length of the growing junction is less than 2 microns. Although satisfying mapping of motifs can be achieved using only 1NN-classifier, these rejection criteria significantly speed up the search process and can also improve specificity (see Fig 2D for a flowchart of the algorithm and S1 File for detailed algorithm description).

### Method validation

**Fig 3:**
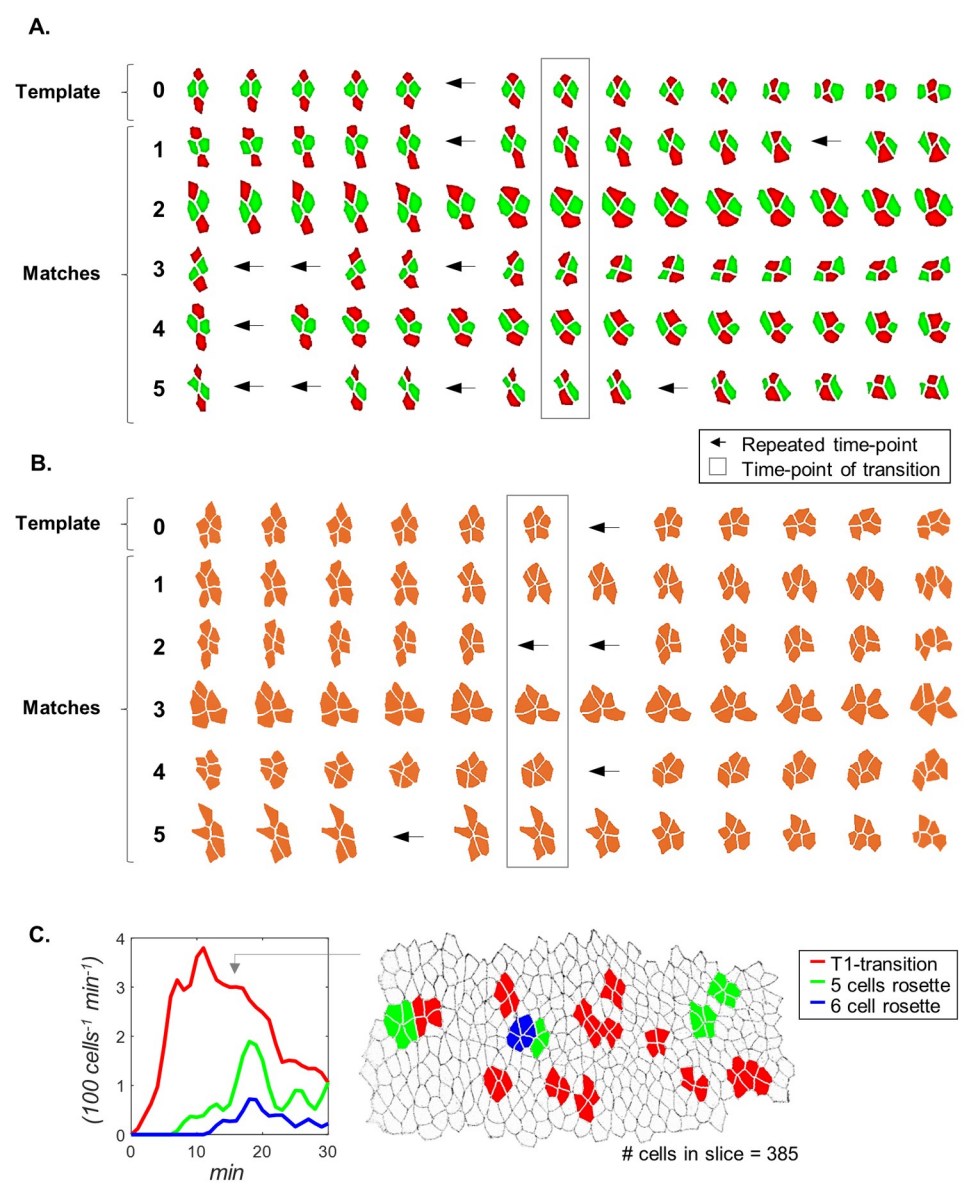
Visualization of detected motif appearances. (A,B) Multiple sequence alignment of identified T1-transitions (A) and 5-cell rosettes (B). Snapshots in the rectangle correspond to the time points of cell rearrangement. Left arrows indicate that the sequence was temporally stretched by repeating the previous time point. (C) Left: Quantification of time-dependent frequencies of T1-transitions (red) and rosettes (green, blue) during the first 30 minutes of GBE, expressed as the number of events occurring in a size normalized tissue of 100 cells over a period of 1 minute. Right: a snapshot taken 16 minutes into GBE, showing the identified motifs, corresponding to cell groups at the transition time point. The image includes other cell groups undergoing rearrangements, but they are not at their transition points.

We validated our motif matching algorithm in three different ways. First, we tested it on synthetic data generated by a computational vertex model of cell rearrangements. This established a sensitivity (true positive) rate of 97.4% and a specificity (true negative) rate of 100% (see S1 File for details). Second, we used the well-established software package “TissueAnalyzer” (TA) as a gold standard for detecting T1-transitions in our acquired live data [40]. Out of 691 validated T1s, TA identified 672 (97.25%) as compared to 674 (97.54%) by our algorithm, thus showing high and comparable sensitivity. However, TA had also identified 45 false T1, as compared to 6 by our algorithm, demonstrating the significantly higher specificity of our approach. In all 45 false T1s detected by TA the cell quartet does manifest both initial and final topologies of T1-transition at two different time points. However, during the intermediate period the cell quartet manifests topologies that are unrelated to T1-transitions, such as the structure of a chain. Such topology related errors are avoided by our approach owing to search space pruning, which rejects any topology that is irrelevant to T1-transition. As distances between cell boundaries in these false T1s are different from a true T1, even without using our pruning mechanism their high DTW dissimilarity scores to the template would have highlighted them as less likely to be T1. Notably, the total runtime dedicated for data preprocessing and T1 detection by TA was 22.5 and 8.5 seconds, respectively, as compared to 135 and 24 seconds by our algorithm, respectively. This difference reflects the rarely avoidable tradeoff between the generality and accuracy of an algorithm and its efficiency. Lastly, as will be elaborated later, by applying our algorithm to imaging data from wild-type embryos, we have successfully recovered previously reported kinetics of cell intercalations: with nonmonotonic rate of T1 formation and delayed appearance of multicellular rosettes (Fig 3, Fig 1 in S2 File, [18]). Following these tests, we proceeded to use our algorithm to analyze the statistical properties of intercalary behaviors.

### Statistical properties of intercalary motifs

**Fig 4:**
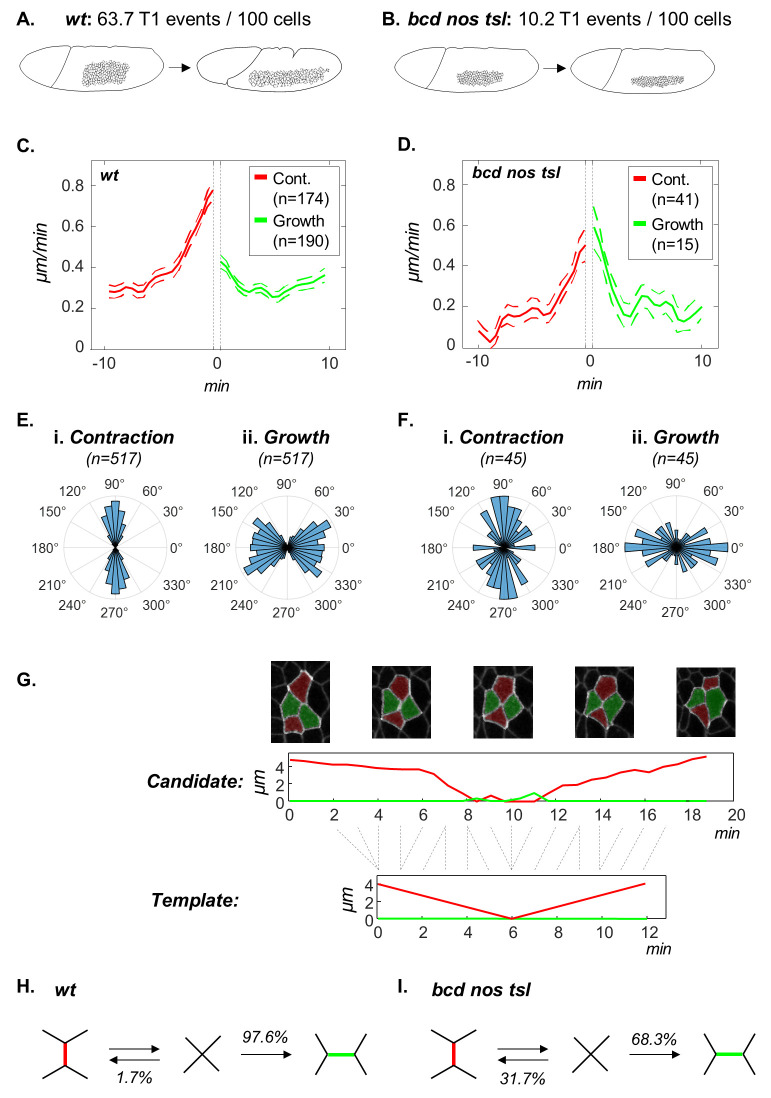
Dynamics and efficiency of T1-transitions. (A,B) The first and last snapshots of GBE imaged in the wild-type (A) and *bcd nos tsl* (B) embryos. Quantification of the average number of T1-transition events within a size normalized tissue of 100 cells throughout GBE shows a significant decrease in their prevalence within the mutant (*p* = 0.0041). (C) The rates of junction contraction (red) and growth (green) in wild-type embryos. Horizontal axis represents the time before contraction completion and after growth initiation. Vertical dashed lines indicate a time gap of between the two events (1.53±1.48 min, see also Fig 2,3 in S2 File). (D) Same as (C) but for *bcd nos tsl* embryos (time gap: 1.53±1.7 min). (E) Rose diagrams showing angle distributions of contracting junctions with respect to the ventral midline (i: 91.70±18.30°; *p* << 0.05 Rayleigh test for circular uniformity), and of growing junctions (ii: 1.20±35.90°; *p* << 0.05). (F) Same as (E) but in *bcd nos tsl* embryos (83.90±41.40°; *p* = 0.011 for contraction; 2.40±41.90°; *p* = 0.012 for growth). 0° = posterior, 90° = dorsal. (G) Bottom: Manually drawn template for a reversing T1-transition. Middle: An example of an identified reversal. Top: Snapshots of the identified reversal. (H) Probabilities of T1 rearrangements and reversals in the wild-type embryos (based on 539 events). (I) Same as (H) but for *bcd nos tsl* embryos (based on 60 events).

In common models of T1 cell rearrangements, contraction of cell junctions is driven by self-amplifying myosin recruitment, which should generate a progressively increasing contractile force on the contracting interface [41]. Working in concert with membrane removal [42], an increasing contractile force should accelerate junction contraction as the T1-transition is approached. The formation of a newly formed interface involves different mechanisms, such as the formation of new cell-cell adhesions [3]. Growth of a newly formed interface should slow down as the cell quartet moves past the transition point and approaches a new stable configuration. To examine these predictions, we mapped T1-transitions in wild type embryos (n = 3; 330 analyzed cells/frame in average), which identified 175±17.6 (avg.±S.D.) high confidence T1s per movie (63.7 T1s/100 cells; see Methods). Because our algorithm automatically extracts the complete lifetime sequence of each event, identification of T1s allows immediate analysis of their temporal dynamics. Indeed, all of these predictions are strongly supported by the rates of contracting and growing cell interfaces (Fig 4C and Fig 2,3 in S2 File): During the last 10 minutes of contraction, the average contracting cell junction nearly triples its contraction rate, while the growing junction reduces its rate by ~30% over the same duration. The average junction contraction and growth rates during these periods agree with previous reports [43,44]. Also consistent with previous studies is the strong alignment of contracting junctions with the short axis of the embryo, and, though to a lesser extent, of growing junctions with the long axis (Fig 4E.i,ii) [45].

The observed remodeling kinetics and orientation are consistent with the reported increase in mechanical tension along myosin II enriched cables established by the AP patterning system [41]. But what happens when these cables are removed? To address this question, we analyzed datasets from embryos devoid of AP patterning information (*bcd nos tsl*). We found that a small number high quality matches to T1-transitions could still be detected in these embryos (avg.±S.D. = 21±2.6 T1s per movie; 10.2 T1s/100 cells; n = 3, 231 analyzed cells/frame in average; Fig 4A and 4B). These residual T1-transitions are likely to be caused by morphogenetic processes that are independent of AP patterning, such as the invaginating mesoderm or cell division [46,47]. The different origin of these residuals is supported by the differences in their orientation and dynamics (Fig 4D, Fig 2,3 in S2 File). In contrast to the strong asymmetry in the kinetics of contracting and growing junctions in wild-type embryos, T1-transitions in *bcd nos tsl* are symmetric around the transition point (*p* = 2.07e-8 and *p* = 1, respectively).

We next asked what fraction of the cell quartets that arrive at a transition configuration actually complete the T1 rearrangement? To answer this question, we scanned the imaging datasets for matches to a different template, one in which the same interface first contracts and then recovers its length. Such a template can be extracted directly from an example provided by the user or, alternatively, supplied as a hand-drawn collection time series that capture the qualitative dynamics of the relevant features (Fig 4G). Our algorithm can handle both formats, but the second one is more convenient when an example cannot be easily found within the dataset. Using this approach, we could readily identify abortive attempts at T1 exchanges (avg.±S.D. = 4.3±1.2 events per movie; 1.1 events / 100 cells; n = 3) and calculate the fraction of total approaches to a T1-transition that result in successful rearrangements. In wild-type embryos, this fraction is 97.6% (Fig 4H., Fig 4 in S2 File; in agreement with [48]). This means that transitions are 41 times more likely to go forwards than backwards. On the other hand, this likelihood is dramatically reduced to 2.2 in bcd nos tsl mutants lacking all AP patterning (Fig 4I). Notably, the average number of T1 attempts (i.e. successful + failed transitions) in the wild type is significantly higher than in bcd nos tsl (68.12 and 14.78 events / 100 cells, respectively; *p* = 0.001, two-sample t-test). This indicates that the reduced number of successful events in the mutant results primarily from the inability to initiate the transition rather than to resolve it. Our analysis suggests that abortive T1s in the wild type initiate similarly to successful T1s but fail due to an interference with a neighboring event. For example, of the 13 reversals in wild-type, 4 were associated with divisions in a neighboring cell and 2 with the invaginating ventral or cephalic furrows. We note, however, that in *bcd nos tsl* resolutions are still slightly biased towards successful T1s. We speculate that this could be in part due to the orthogonal mesoderm invagination.

**Fig 5:**
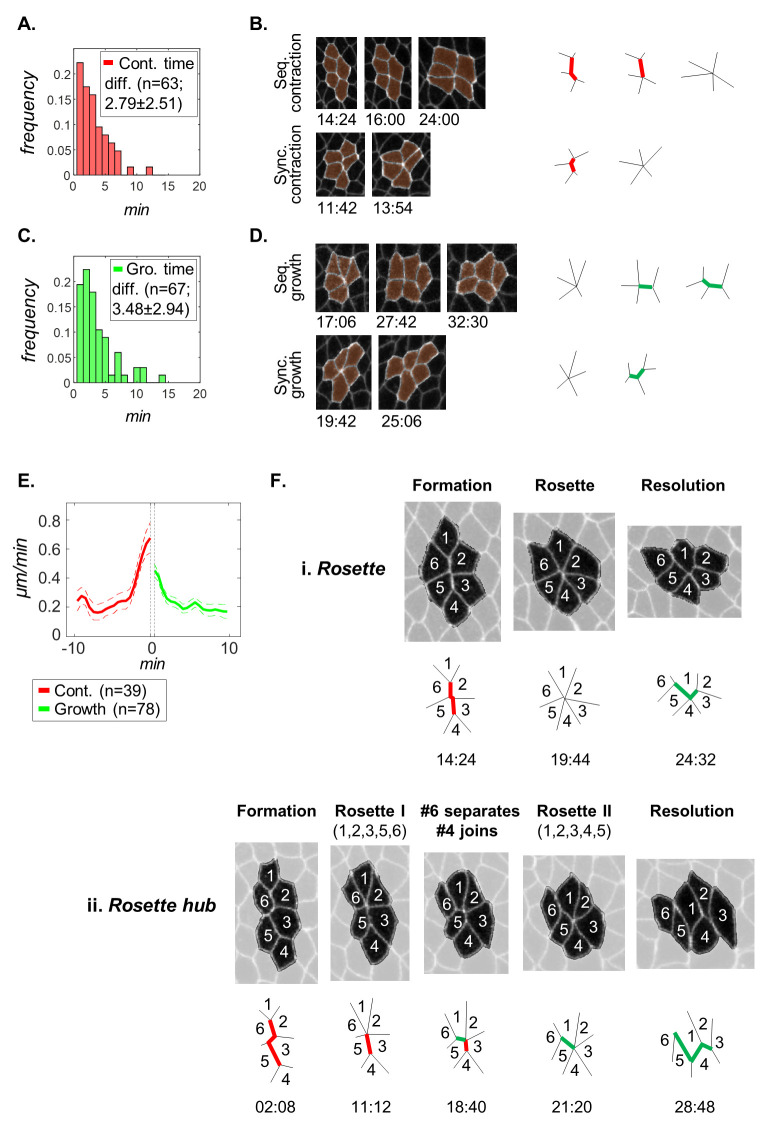
Analysis of rosette dynamics. (A) Distribution of time difference between contraction of the two disappearing junctions in 5-cell rosettes. (B) Left: Snapshots illustrating synchronous and sequential formation of 5-cell rosettes. Right: schematics of junction dynamics. (C,D) Same as (A,B), respectively, but for growing junctions. (E) The rates of junction contraction (red) and growth (green) during the formation of 5-cell rosettes. The horizontal axis is the time before contraction completion and growth initiation (vertical dashed lines indicate a possible time gap between the two events). Average contraction rate is significantly lower than for T1-transition. (F) Schemes of a rosette (i) and a rosette hub (ii). Top: snapshots throughout the lifetime of the motif. Cell numbering allows relating cells over time. Bottom: a simplified diagram of the exemplified motif. A rosette hub (ii) initiates with the formation of a rosette, followed by an iterative process, whereby at least one cell separates from the rosette (cell #6), followed by the joining of at least one new cell (cell #4), all the while maintaining at least 4 cells surrounding the core vertex (cells #1,2,3,5). As a result, the central vertex serves as a hub for intercalating cells.

Contrary to T1-transitions, the formation of rosettes requires removal of multiple cell interfaces, which creates alternative pathways for cell rearrangements. For instance, with two contracting junctions, 5-cell rosettes can reach the single vertex state either sequentially, similarly to formation of a T1-transition followed by inclusion of a fifth cell, or simultaneously. To examine these possibilities, we mapped 5- and 6-cell rosettes in wild type embryos (avg.±S.D. = 66.7±12.0 and 27.6±8.5 events per movie, 18.0 and 7.3 events / 100 cells, respectively; n = 3). Our statistical analysis indicates that most rosettes form in a sequential manner, where in 95% of all cases the interval between the first and last junction disappearance is 7 minutes or less (Fig 5A and 5B). This observation is also true with respect to rosette resolution (Fig 5C and 5D). The sequence of multiple contractions and resolutions governing rosette morphology span more than 10 minutes and contrasts with the relatively short durations four-way vertices during T1-transitions (1.53±1.48 minutes, n = 526). Interestingly, our inspection of rosettes revealed a deviation from the simple scheme of *convergence* (*vertical junctions contract)* →*rosette structure (all cells meet at a single vertex)* → *extension (horizontal junctions form and elongate)*. Instead, the rosette structure stage is replaced by the resolution of one or two members of the rosette, followed by the joining of one or two new cells while maintaining a core of at least four cells (Fig 5Fi,ii). We found that 11% of all 5-cell rosettes and 16% of all 6-cell rosettes form and resolve in this way, indicating that this newly identified mode of intercalation is not uncommon in GBE. Since rosette hubs cannot be explained according to the existing definitions of T1-transitions and rosettes, these results call for the reevaluation of the basic building blocks underlying GBE. Thus, our algorithm provides a systematic approach for mapping of known motifs and guides the discovery of new behaviors.

## Discussion

Advancing the modular view of morphogenesis, according to which tissue-scale processes are assembled from a small number of motifs, requires efficient algorithms for motif mapping in live imaging datasets. Most existing algorithms for this task use rigid criteria for graphs of cell connectivity [18–25]. Such criteria typically have immediate interpretations and can be implemented with little effort. However, they are not easily generalized to analyze temporal sequences or the distribution of molecular factors within the tissue. In our strategy, the motif does not have to be formally defined. Instead, a motif template is provided as a time series extracted from a user selected example. We illustrated our strategy by constructing ensembles of high confidence matches to T1- and rosette rearrangements, paving the way for their statistical analysis. This analysis recovered several of already known aspects of convergence-extension movements, such as high efficiency of T1-transitions and sequential appearance of T1s followed by rosettes [18,48,49]. At the same time, our analysis also demonstrates that rosette assembly and resolution are sequential processes, which has led us to the identification of a new mode of iterative rosette intercalation, to which we refer as rosette hubs.

Time-series subsequence matching have been long acknowledged as a flexible and robust approach for template-based search in molecular sequence databases and dynamic gene expression patterns [50,51]. In the context of live data of evolving tissues, time series approaches have been used for detecting and phase labeling cell division events [52]. However, these earlier approaches were designed to search datasets containing independent items and thus cannot handle the complex connectivity of cell sheets. To bypass this obstacle, we transform our live data from an attributed dynamic graph into a multidimensional sequence dataset. This transformation immediately allows us to utilize the broad arsenal of tools developed by the time-series data mining community.

Our approach holds several fundamental advantages over existing algorithms. First and foremost, the ability to combine topological organization, geometric and molecular attributes of cells as time series dimensions makes our algorithm suitable for mapping a broad range of sub- to multi-cellular behaviors. This is in contrast with mapping behaviors that are associated directly with adjacency relations, as traditionally done. We demonstrate this capacity by extracting completing and reversing T1-transitions and multicellular rosettes. Second, subsequence matching using DTW provides a natural means for approximating the complete time range of each event. Moreover, it can be used for labeling different phases throughout the lifetime of the motif labeling (see S3 File—Automated mapping and phase labeling of dividing nuclei and ingressing neuroblasts). As our method validation indicates, classifying motifs based on temporally evolving patterns rather than updates in topology alone significantly increases robustness against unstable T1s. And last, the ability to search a live image by pointing to an example rather than formulating quantitative criteria significantly relaxes the need for technical knowledge on the user’s side. As our mapping of reversing T1s demonstrates, in the absence of a concrete example, accurate mapping and analysis of behaviors can still be achieved by manually drawing the presumed dynamics. Notably, despite its demonstrated generality and accuracy, our algorithm is not without its handicaps. To guarantee suitability for a broad range of sub- to multi-cellular motifs, our algorithm requires an exhaustive search for all connected induced subgraphs, followed by a large volume of sequence alignments. Taken together, these compromise its efficiency as compared to existing methods. Although our reported runtimes are well within reason for the typical dataset, future improvements would better align its performances with the rapidly approaching in-toto studies.

In a broader view, our work demonstrates the potential of a time series data mining approach in studying tissue morphogenesis. The discovery of repeating behaviors in a morphogenetic process is the first step toward deciphering how cells organize to result in tissue level deformations. So far, this had been done by meticulous visual inspection, which is bounded by the complexity of the data and the inherent biases of our perception. Relying on our time series representation, these explorations could be done using de-novo motif discovery algorithms, in which a sequence dataset is searched for statistically overrepresented segments in a fast, systematic, and unbiased manner [53,54]. Such modular decomposition approaches proved to be transformative in dealing with large volumes of data from sequencing and structural studies of DNA, RNA, and proteins [55–57]. In the future, this could serve as the basis for recovering a complete dictionary of tissue behaviors. We believe that these decompositions are essential for revealing the mechanisms by which diverse morphogenetic motifs coordinate their actions and synergize with each other as embryos evolve from relatively simple initial conditions to progressively complex structures.

## Methods

### Fly stocks and genetics

Control flies are the F1 offspring of the following cross: ♀ w; +; ru klar His2Av-GFP sqh-Gap43::mCherry / ru klar His2Av-GFP × ♂ w; +; ru klar. *bcd nos tsl* flies were generated starting from the following cross: ♀ w; +; ru klar His2Av-GFP sqh-Gap43::mCherry bcd^E1^ nos^BN^ e tsl / TM3 Sb × ♂ w; +; bcd^E1^ nos^BN^ e tsl / TM3 Sb. From F1 offspring of this cross: w; +; ru klar His2Av-GFP sqh-Gap43::mCherry bcd^E1^ nos^BN^ e tsl / bcd^E1^ nos^BN^ e tsl females were crossed with Ore-R males. Embryos resulting from this cross (overall F2) were used for *bcd nos tsl* analysis. Fly for analysis of nuclear cleavages (S3 File) is a wild-type expressing H2AV::mRFP. All embryos used in this study were grown at either 18 or 25°C and imaged at 20°C.

### Live imaging

Embryos were dechorionated by gently rolling them on a double-sided adhesive tape until the chorion layer was completely peeled. Then, embryos were mounted in a halocarbon oil 27 (Sigma) on a gas-permeable membrane (aka bio-foil; Kenneth Technologies), and covered with a high-definition 1.5H coverslip (Paul Marienfeld). Images were acquired 5–10 *μm* below the visible apical end of the cells on a Leica SP5 confocal system with an HCX PL APO CS 63×/1.4-NA oil-immersion objective. Time-lapses were acquired at 561 *nm* excitation wavelength, line averaging with 1–3 repeats, frame taken every 29–32 seconds, and pixel size 0.2405 *μm^2^*. Embryo for live image of nuclear cycles (S3 File) was dechorionated with 50% bleach, washed and mounted on Biofoil membrane in halocarbon 27 oil. The embryo was then imaged on the Nikon Ti-E microscope with the Yokogawa spinning disc (CSU-21) module using the 561 laser to visualize nuclei at 10 s intervals.

### Image segmentation, object classification and cell tracking

Segmentation for cell boundaries was done for each frame independently using the “Autocontext” and “Object classification” workflows in the publicly available software Ilastik [58]. The classifier was trained jointly on time-lapses of one wild-type and one bcd nos tsl embryos, then applied on all analyzed time-lapses. This resulted in binarization of all the pixels within each slice into “membrane” or “cell”. To fine tune the detection of the membrane’s central line and thin inter-cellular gaps to 1 pixel we first used morphological reconstruction to modify *I* so that it only has local minima where *B* pixels are set. This operation ensures that upon execution of watershed transformation, only set pixels in B will serve as seeding points and thus avoid creating new cells that were not a part of the manual segmentation. To complete the segmentation, we applied a watershed transform (pixel connectivity = 4). All live movies were thoroughly inspected and corrected manually for errors in segmentation.

To perform cell tracking, we implemented an image registration (IR) based tracking algorithm. In this approach cells in the binary (i.e. segmented) slice at the first time point are indexed arbitrarily. Then, similarly to a mathematical induction, by inferring the spatial correspondence between slices at consecutive time points cell indices can be propagated from one time point to the next. To perform the spatial matching we used Maxwell’s demons algorithm for non-rigid registration [59] (matlab implementation of “imregdemons” with three pyramid levels, for which the number of iterations was: [10,20,30]). Since the binarized slices are relatively poor in spatial information, the matching was calculated on the raw microscopy slices and applied on the binarized slices. Given a slice at time point t with indexed cells and the a non-indexed slice at time point t+1 which had been registered to the former and, we determine the index of a cell according to the following criterion: If there exists a cell at time point t such that the area of overlap between the two cells is more than half the area of each of the two cells, we propagate the cell index from t to t+1. Otherwise, we conclude that this cell has entered the region of interest between time points t and t+1, and assign it with the index: max ({*all cell indices*})+1. Notably, a thorough visual inspection of all processed live images did not detect any errors in tracking (see S1 File for visual demonstrations).

### Membrane polygonization and construction of adjacency matrix

Following image segmentation and tracking the geometry of the membrane network was converted from image representation to a vertex model. As a result of the watershed we applied at the end of the segmentation, the collection of membrane pixels in each slice can be regarded as the skeletonization of the membrane region. To turn the skeleton into a vertex model we first scan the frame in search for skeleton branch-points, which correspond to tissue vertices (i.e. meeting points of 3 cells or more). These branch-points allow us to identify each individual edge as the chain of connected pixels between two branch-points. One way to turn a pixel represented edge into an edge in a vertex model is by defining a straight line between the two branch points of the edge. However, this approximation will ignore the curvature of curved edges and therefore underestimate their true length. Instead, we approximate each edge as a chained sequence of straight lines that pass through the centroids of all pixels in the edge (red line in the figure below). To reduce data volume and give edges a more natural smooth appearance we reduced the number of line fragments using the Douglas-Peucker line simplification algorithm with a toll of 1 pixel (equal to 0.2405 microns). Based on this vertex model representation of the tissue we can now define the binary adjacency matrix of the cells as equal 1 if and only if the two corresponding cells share an edge (a shared vertex does not qualify cell adjacency; see S1 File for visual demonstrations).

### Quantification of motif frequency

We define motif frequency as the number of appearances of the motif within a size normalized tissue of 100 cells per minute. The time of appearance of an intercalary motif is defined as the time point at which all cells meet at a single core vertex. For simplicity, assume we wish to quantify the frequency of T1-transitions within a single frame containing 300 cells, in which 10 T1 events have been identified by our algorithm. Estimating the number of events per cell as: 10/300 (i.e. #events/#cells) is likely to result in underestimation, since cells overlapping with the boundary of the frame (~75 cells = 25%) can potentially participate in T1 events with cells outside of the frame which cannot be discovered. Following the same rationale, estimating the number of events per cell as 10/(300–75) (i.e. # events / # non-boundary cells) can result in overestimation, due to inclusion of T1 events that are only partially within the set of non-boundary cells. To avoid these issues, we first calculate for each appearance of T1 the fraction of its cells that are non-boundary cells. For instance, a T1 at the center of the frame will be counted as 1, since all its cells are non-boundary cells, while a T1 in which only three of its cells are non-boundary cells will be counted as 3/4. Then, we divide the sum of calculated fractions by the number of non-boundary cells: $\frac{\frac{1}{4}\sum_{a_{i}\in A}\left|a_{i}\cap C\right|}{\left|C\right|}$, where *A* = {*a*_*i*_} is the set of all appearances of T1-transitions identified by our algorithm in this frame, $a_{i}=\{c_{i}^{1},c_{i}^{2},c_{i}^{3},c_{i}^{4}\}$ is the set of four cells from which event *a*_*i*_ is assembled, and *C* is the set of all non-boundary cells within the frame. Since the value we calculated so far reflects the number of events per cell within a time window equal to the inter-frame interval (*dt*), we convert the values to account for a time interval of one minute by dividing the calculated value by *dt*. Lastly, to shift the resulting value to a more intuitive range we multiply it by 100, to represent the estimated number of events within a square tissue of 100 cells.

### Quantification of junction kinetics

To describe the process of T1-transitions based on the contracting interface, we calculate the length of the relevant junctional interface at a given time point as the sum of lengths of all linear segments assembling the junction in the polygonised membrane (see Membrane polygonization). The sequence of junction length over time is assembled by calculating the length independently at each time point throughout the time range of the motif. Time points in a T1 progression when the contracting interface no longer exists and new interface has not yet formed were discarded by truncating the sequence from the earliest time point in which the junction reaches zero length to the last time point before a new junction initiates persistent growth. This procedure applies also to wobbly T1s, wherein the transition period involves short transient formations of DV or AP interfaces. Since junction length is influenced by periodic pulses of medial myosin, we smooth the sequence using a moving average filter of width 3 (~1.5 minutes). Since different movies were acquired at different temporal sampling rates, we standardize the sampling rate of calculated sequence to Δ*t* = 30 seconds using linear interpolation. Lastly, we calculate the rate of contraction as Δ*l*/Δ*t* for every pair of consecutive time points. To calculate the average contraction rate over all junctions we extract from each sequence the last 10 minutes (i.e. 20 time points) preceding the disappearance of the junction and discard all sequences that persist for less than 10 minutes. In 5-cell rosettes we apply the same procedure independently to each of the two contracting and two elongating junctions.

### Quantification of junction orientation with respect to the ventral furrow

First, 3–4 points were manually marked along the ventral midline in every 10th slide. Then, a 3rd or 4th degree polynomial of the form *VF*(*x*) = ∑_*i* = 0,…,4_*a*_*i*_*x*^*i*^ (in the case of a cubic polynomial *a*_4_ = 0) was fitted independently to the marked points within each slide, thereby overlapping with the line of the ventral furrow. Interpolation of ventral furrow curves in the remaining slides was done by independently interpolating {*a*_0_,…,*a*_4_} using pchip interpolation. The angle between an edge passing through the points {(*x*_0_,*y*_0_), (*x*_1_,*y*_1_)} and the ventral midline *VF*(*x*) in a given time point was calculated as: $\left|\tan^{-1}\;VF^{\prime }(\tilde{x})-\tan^{-1}\;\left(\frac{y_{1}-y_{0}}{x_{1}-x_{0}}\right)\right|$, where $\left(\tilde{x},VF(\tilde{x})\right)$ is the closest point to $\left(\frac{{x_{0}+x}_{1}}{2},\frac{{y_{0}+y}_{1}}{2}\right)$ on the ventral midline. For the purpose of generating a radially symmetric distribution plot, for each calculated angle α we added the value α+180^0^.

### Statistics

Testing for differences in prevalence of T1-transitions per 100 cells between wild-type and bcd nos tsl embryos (Fig 4A) was done using two sample one tailed t-test (c.i. = [36.4, ∞), test stat. = 8.38, dof = 2.37). Testing for non-uniformity in junction orientation (Fig 4E and 4F) was done using Rayleigh test [60] with Bonferroni correction for multiple hypothesis testing. Since junction orientations have a perfect 2-fold symmetry, as a preprocessing step all angle values were multiplied by 2. Testing for differences in average junction length change rate were carried using one-way ANOVA, followed by Tukey’s HSD post-hoc test of significance.

### Software

All parts of the pipeline except for the implementation of the algorithm for generating subgraph induced connected components of size k, and all following analyses, were developed in matlab 2018a [61]. For basic image processing tasks such as rotation, ROI cropping and format conversion we used Fiji [62]. Image segmentation and object classification were done using Ilastik [58]. Implementation of the algorithm for generating subgraph induced connected components of size k was done in c language by Shant Karakash and Berthe Choueiry (University of Nebraska-Lincoln, NE, USA) and used under their permission.

### Hardware

Code for the described pipeline was developed on a Dell PowerEdge R930 server carrying four Intel(R) Xeon(R) CPU E7-4850 v4 @ 2.10GHz with 16 cores each and 2TB RAM, with RedHat OS. Cell and nucleus segmentation and tracking, and all the analyses of the extracted motifs were done on Intel(R) Xeon(R) CPU E5-1620 v4 @ 3.5GHz, with 32GB memory and Windows 10 64 bits OS.

## Supporting information

S1 File Algorithm description. (DOCX)

S2 File Additional statistical analyses of the dynamics and temporal distribution of T1-transitions and rosettes. (PPTX)

S3 File Template-based mapping and phase labeling of dividing nuclei and ingressing neuroblasts. (DOCX)
