# Supplementary material for: Template-based mapping of dynamic motifs in tissue morphogenesis
Source: PLoS Comput Biol. 2020 Aug 21;16(8):e1008049. doi: 10.1371/journal.pcbi.1008049 (PMC7442231; doi:10.1371/journal.pcbi.1008049)
Supplement: S1 File — (DOCX) [file pcbi.1008049.s001.docx]

**Supplementary file 1 – Algorithm description**

**Described steps:**

1. Movie preprocessing:
   1. Cell segmentation and tracking
   2. Membrane polygonization
2. Algorithm input: template definition and selected features
   1. Available features
3. Algorithm:
   1. Generation of all connected induced subgraphs of size k
   2. Transformation of tissue into time-series data
   3. Candidate search by sub-sequence matching
      1. Cell pairing in multicellular motifs
   4. Accuracy refinement using nearest-neighbor classification
   5. Early and late candidate rejection
   6. Rule based candidate filtering
4. Algorithm output:
   1. Automated propagation of temporal labeling
5. Benchmark analysis using vertex model simulation
6. Literature

**MOVIE PREPROCESSING:**

**Cell segmentation and tracking:**

Segmentation for cell boundaries was done for each frame independently using the “Autocontext” and “Object classification” workflows in the publicly available software Ilastik [1]. The classifier was trained jointly on time-lapses of one wild-type and one bcd nos tsl embryo, then applied on all analyzed time-lapses. This resulted in binarization of all the pixels within each slice into “membrane” or “cell”. To fine tune the detection of the membrane’s central line and thin inter-cellular gaps to 1 pixel we first used morphological reconstruction to ensure that local minima in the raw image are present only where the binarized image is set to “cell”. This operation ensures that upon execution of watershed transformation, only “cell” pixels in will serve as seeding points and thus avoid creating new connected components that were not a part of the manual segmentation. Lastly, we applied a watershed transform (pixel connectivity = 4). All live movies were thoroughly inspected and corrected manually for errors in segmentation. The figure below shows an overlay of a region of interest from a raw confocal slice (grayscale) with the resulting segmentation procedure (red).

To perform cell tracking, we implemented an image registration (IR) based tracking algorithm. In this approach cells in the binary (i.e. segmented) slice at the first time point are indexed arbitrarily. Then, similarly to a mathematical induction, by inferring the spatial correspondence between slices at consecutive time points, cell indices can be propagated from one time point to the next. To perform the spatial matching we used Maxwell’s demons algorithm for non-rigid registration [2]. For this we used the Matlab’s “imregdemons” function using three pyramid levels and [10,20,30] iterations in levels 1-3, respectively. Since the binarized slices are relatively poor in spatial information, the matching was calculated on the raw microscopy slices and applied on the binarized slices. Given a non-indexed slice at time point t+1 that was registered to the indexed slice at time point t, we determine the index of an unindexed cell according to the following criterion: If there exists a cell at time point t such that the area of overlap between the two cells is more than half the area of each of the two cells, we propagate the cell index from t to t+1. Otherwise, we conclude that this cell has entered the region of interest between time points t and t+1, and assign it with the index: $max(\left\{ all currently assigned indices \right\})+1$. Notably, a thorough visual inspection of all processed live images did not detect any errors in tracking. The figure below demonstrates the results of applying non-rigid registration between two consecutive time points (top), and the result of propagating cell indices between the corresponding two slices.


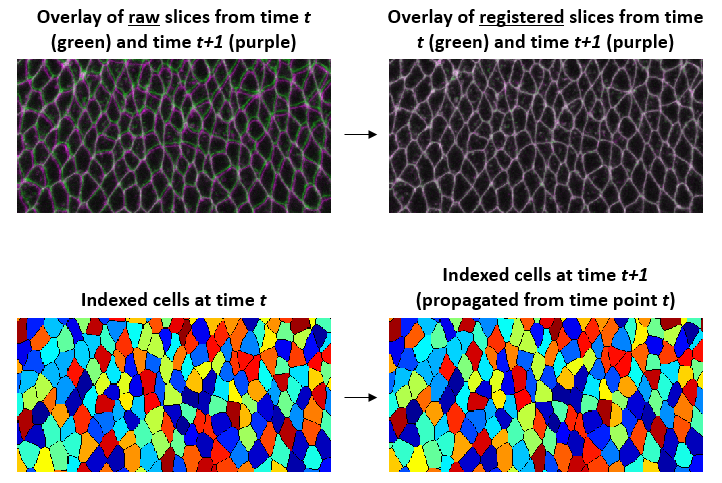


**Membrane polygonization**


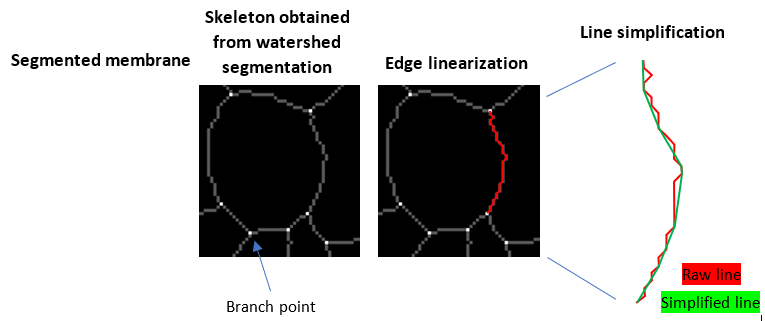
Following image segmentation and tracking, the geometry of the membrane network was converted from pixel representation to a vertex model. As a result of the watershed we applied at the end of the segmentation, the collection of membrane pixels in each slice can be regarded as the skeletonization of the membrane region. To turn the skeleton into a vertex model we first scan the frame in search for skeleton branch-points, which correspond to tissue vertices (i.e. meeting points of 3-8 cells; white isolated pixels in the figure below). These branch-points allow us to identify each individual edge as the chain of connected pixels between two branch-points (gray pixels in the figure below). One way to turn a pixel represented edge into an edge in a vertex model is by defining a straight line between the two branch points of the edge. However, this approximation will ignore the curvature of curved edges and therefore underestimate their true length in downstream analyses. Alternatively, one can represent each edge as a chained sequence of straight lines that pass through the centroids of all pixels in the edge (red line in the figure below). However, this approach is known to overestimate the true edge length. Therefore, we reduced the number of line fragments using the Douglas-Peucker line simplification algorithm with a tol of 1 pixel (equal to 0.2405 microns; green line in the figure below). The resulting edge lengths using this tol value are in good agreement with manually calculated edge lengths (data not shown). Based on this vertex model representation of the tissue we can now define the binary adjacency matrix of the cells as equal 1 if and only if the two corresponding cells share an edge (a shared vertex does not qualify cell adjacency).

**ALGORITHM INPUT: TEMPLATE DEFINITION AND SELECTED FEATURES**

To search a motif within a tissue the algorithm requires an example of the searched motif and the names of the features based on which the template and the analyzed candidates will be represented.

Template definition can either be “example based” or “manually drawn”:

- *Example based* template: In this mode the user manually selects a single example of the searched motif from the analyzed movie to be used as a template. To point to the selected template the user provides the algorithm the indices of all k cells that compose the template, as well as the indices of the first and last frames in which the template appears within the movie. For example: *cell_indices = [7,18,90,115], time_range = [10,25]*. We used this option to search for T1-transitions, 5- and 6-cell rosettes and nuclear cycles.
- *Manually drawn* template: In case the user would like to specify a template without using a real motif example from the tissue, it is also possible to provide a time-series (i.e. the curves of the features) that was either drawn manually or programmatically. We used this option to search for T1-reversals.

**Available features:**

The algorithm currently provides three types of features:

1. **Single-cell features:** ~200 different features that describe the geometry (e.g. area, circumference, aspect ratio etc.) and pixel intensity distribution (average, maximal, minimal, kurtosis of intensity etc.) of each individual cell regardless of its environment. Most of these features were calculated by the “Object classification” module in Ilastik.
2. **Cell-pair features:** 5 features that describe relations between a pair of cells. These are:
   1. *junction_length* (if cells are not neighbors then the value is 0).
   2. *junction_length_deriv* (the first derivative of *‘junction_length’*).
   3. *cell_boundary_distance* (this is the minimal distance between the contours of two cells. This was calculated as the minimal Euclidean distance between all pairs of vertices from the two polygons and all pairs of vertex and edge, calculated independently for each time point).
   4. *cell_boundary_distance_deriv* (the first derivative of ‘*cell_boundary_distance’*).
   5. *are_neighbors* (Boolean, equals 1 iff *junction_length* > 0).
3. **Statistical summary features:** wherein a statistical operator like minimum, maximum, mean or standard-deviation is applied on all values of any of the ‘single-cell’ or ‘cell-pair’ features at each time point.

For the analyses described in the body of the manuscript we used the following features:

- T1-transition and T1-reversals: *cell_boundary_distance*. Since here k=4, the total number of features is $\left( \begin{matrix} 4 \\ 2 \end{matrix} \right)=6$. The advantage of this feature over the *junction_length* feature in describing T1-transitions is that the only non-zero values at any given point are either the distance between the A and P or between the D and V cells. As a result, no noise is added to the DTW matching by the length of junctions between neighboring cells.
- 5- and 6- cell rosettes: *maximum(cell_boundary_distance)*. As this is a statistical summary feature, the total number of features is 1.

**ALGORITHM:**

**Generation of all connected induced subgraphs of size k:**

To allow the algorithm to search the motif exhaustively within the tissue, the list of all connected components (CC) of k cells must first be extracted from the movie. Specifically, by ‘connected component’ we refer to a group of cells in which every cell can be reached from every other cell by traveling only through neighboring cells from that group.

To do so, we take a graph-theoretic approach, starting by calculating the dual graph of the polygonised membrane generated earlier (i.e. the cell-adjacency matrix). In graph theoretic terms the task of extracting k-cell CCs is referred to as “generation of all connected induced subgraphs of size k”. For this we use the fast implementation of the algorithm in [3,4] (courtesy of Berthe Y. Choueiry and Shant Karakashian from the University of Nebraska-Lincoln). Lastly, we calculate for each identified CC the list of the time points in which it exists. From this point on, a CC tracked over time will be referred to as a candidate.

The following is an example of the output when k=4 using Matlab notation:

CC index Cell indices Time points

[1] [1,3,11,24] [3:10, 12:50]

[2] [1,3,78,130] [15:52]

…

In the example above CC #1 exists over two separate time ranges: from time point 3 to 10 and from time point 12 to 50.

The following table provides the average number of cells and identified candidate sequences for k = 4,5,6 per movie, for both wild-type and *bcd nos tsl* embryos (in both n=3):

|  | # cells in tissue | # 4-cell neigh. | # 5-cell neigh. | # 6-cell neigh |
| --- | --- | --- | --- | --- |
| Wild-type | 320 | 42,048 | 206,936 | 1,029,276 |
| *bcd nos tsl* | 231 | 11,291 | 47,386 | 204,281 |

**Transformation of tissue into time-series data:**

To prepare the tissue for subsequence matching, a time-series is constructed independently for each candidate. This multidimensional vector contains the values of the selected features for all participating cells at every time point. E.g. for a candidate of four cells (k=4), which exists for 10 time points and the selected feature is cell area, the feature vector will be of size 10×4. To standardize the scores of the matching and to allow combining features with different dimensions (e.g. a description based on both cell area and cell circumference), each calculated feature is normalized using Z-score, resulting in a range of values with mean = 0 and S.D. = 1.

**Candidate search by sub-sequence matching:**

At this point the dynamics of the phenotypes of the template and all candidates are represented as time series data. In this formulation, appearances of the motif can be discovered by matching the sequence of the template against the sequence of each candidate and identifying subsequences that show high similarity to the template. To perform subsequence matching we used Dynamic Time Warping (DTW). Figuratively, given a template sequence and a candidate sequence, DTW finds a sub-sequence of the candidate that is most similar to the template, while allowing both the template and the matched subsequences to be stretched nonlinearly in order to optimize the match by repeating time points. The figure below provides a simplified illustration of the calculation. First, a warping matrix is calculated, wherein entry *(i,j)* contains the distance (i.e. dissimilarity) between the value of the template at time point *i* and the value of the candidate at time point *j*. After the matrix is calculated, the algorithm finds a path spanning over the entire length of the template (i.e. from bottom to top), that minimizes the sum of all traveled cells. Thus sum is referred to as the dissimilarity score between the template and the matched subsequence. The traveled path is referred to as the warping path, and its length is the warping length, which indicates the total number of time points in the template and in the matched subsequence after having being warped to one another (for a mathematical description see [5]).


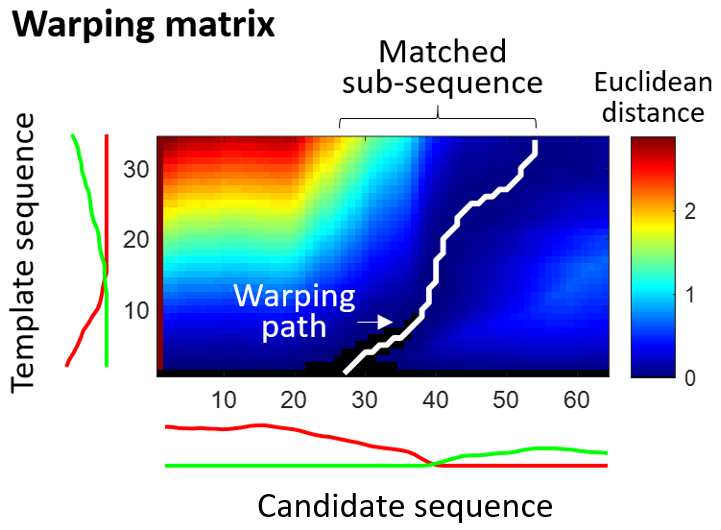


The common metric used for the distance between pairs of time points is the Euclidean distance. Since the dissimilarity score is calculated as the sum of Euclidean distances along the entire warping path, the dissimilarity score increases with the length of the warping path. To allow estimation of the quality of the match regardless of its length we normalize the dissimilarity score by the length of the warping path.

***Cell pairing in multicellular motifs:*** Since cells in multicellular motifs can have distinct roles and thus different features, the detection of similar subsequences therefore relies on proper matching between the cells. For instance, when searching for a T1-transition, the A and P cells of the template must be paired with the A and P cells of the matched candidate. Since the identities of the cells in a given candidate are not known in advance, we ensure the detection of an appearance of the motif by considering all k! permutations template cells. The subsequences derived from a candidate are therefore the union set of all identified subsequences in all k! permutations. This is implemented by pre-calculation of all k! permutations of the features within the time-series of the template. For a visual description see the figure below.

Figure caption: (A) A time-series of confocal images of the template in a search for T1-transitions. Each cell is color coded for visual assistance. White asterisk marks the time point of transition. (B) Left: the index of each cell within the image of the tissue. Right: the features within the feature vector of the template is consisted from all six pairwise *cell_boundary_distance*, and can be ordered in 4!=24 cell permutations. Permutations #1,7,8,24 are shown. Note that due to the symmetry of the *cell_boundary_distance* feature permutations #1,24 are identical. (C,F) The feature vectors of two inspected candidates from the analyzed tissue and the matched template overlaid in red. Note that different permutations of the template are suitable for different quartets. (D,G) Temporal alignments of the template to each of the identified T1s from C,F resulting from the application of the DTW. Empty frames with left arrows mark that the time point to the left is replicated to account for differences in temporal dynamics (similarly to a ‘gap’ in DNA sequence alignment). In addition to allowing to find the motif within the tissue, the automated pairing of cells allows to propagate the identity (color) of each cell from the template to the candidate, and the temporal alignment allows to automatically propagate the time points of significant events from the template to the candidate, such as the time point of transition (see white asterisk), therefore simplifying later analyses. (E) Illustration of correct pairing (left) between two quartets of cells in the beginning of a T1-transition, wherein the nearing cells are paired to the nearing cells and so are the parting cells, and incorrect pairing (right), wherein the nearing cells are paired to the parting cells and vice-versa, which results in poor alignment between the corresponding features.


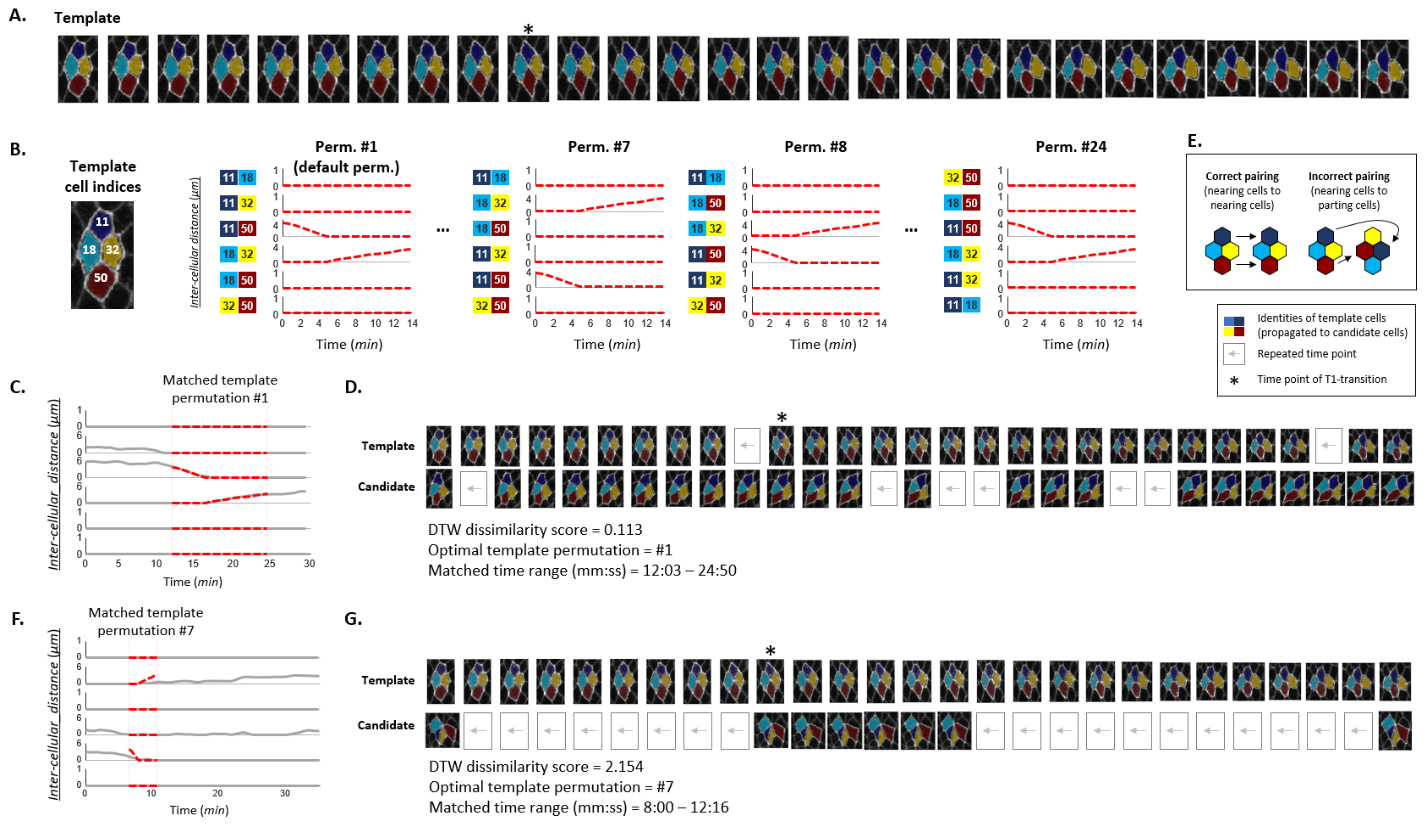


**Accuracy refinement using nearest-neighbor classification:**

As DTW can return multiple matched subsequence from each candidate, most candidates are likely not to be true appearances of the motif and should therefore be screened out. Ideally, a single threshold applied on the dissimilarity scores would separate true candidates from irrelevant tissue movements. However, when the studied motif is not sufficiently distinguishable from the remaining repertoire of the tissue, a single threshold will not be reliable and result in high false positives and/or false negatives. Therefore, to automatically and accurately screen for true hits we implemented a supervised learning classifier based on a 1-Nearest Neighbor (1NN) rule, wherein the label of the candidate is copied from the most similar sequence within a pre-labeled training set.

Construction of a training set by manually marking true and false appearances of the motif directly from the unprocessed time-lapse can be highly time consuming. Therefore, the default training set of a newly defined motif classifier is the empty set, and it is populated interactively with examples from the analyzed tissue as the user labels more candidates as ‘true’ or ‘false’ to refine the classification, until satisfying accuracy is achieved. Importantly, this training set can be used for all future searches of the motif, and in case of need can be updated by changing the labels of training set examples or adding new ones.

Below is a screenshot from a graphical user interface that was written for the 1NN classifier:

Top: time point matching between the training-set sequence (37 time points) and a candidate sequence (28 time points). The alignment was split into three rows as it is too long to be displayed in a single row within the limited computer screen. Similar to DNA sequence alignment, matched time points are aligned vertically. Missing time points in either sequence indicate that the algorithm had stretched to optimize the match by repeating the time point to the left.

Bottom:

- “Motif” and “Not” panels: sequences that were automatically classified as T1s or not T1s, respectively. Allows the user to inspect the alignment of each classified candidate to the training-set sequence.
- “Class” panel: allows the user to correct the classification of the selected candidate.
- “Match info” panel: provides information on the indices of the contained cells and the first and last time points of the candidate and the training set sequences.
- “Training set” panel: allows the user to select a sequence from the training set and see which candidates were classified based on it.
- “Labeled candidates” panel: shows the list of candidates that were classified based on the selected “Training set” sequence (populated only after the selection).


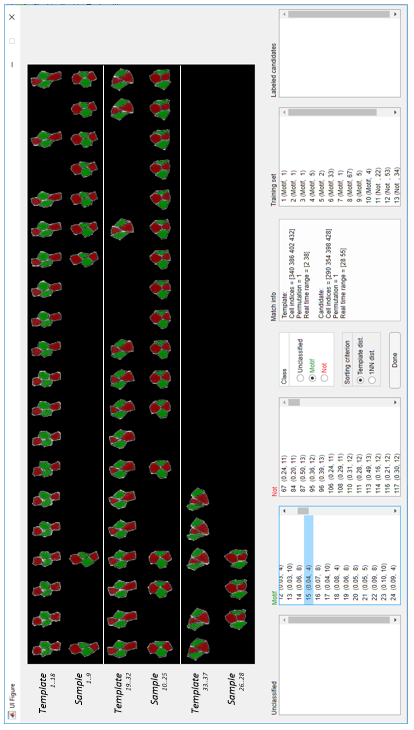


**Early and late candidate rejections:**

A central challenge in an exhaustive search for a motif within a typical size tissue is the large number of sequence comparisons required throughout the entire pipeline. For instance, a plausible search for T1-transitions in a time-lapse of the ventrolateral ectoderm during the fast phase of GBE includes approximately 320 cells over 56 time points, giving rise to ~42,000 4-cell candidates. Multiplied by 4! = 24 cell-to-cell matching possibilities to the template results in ~10^6^ sequence matchings. The initial candidate search results in an average of ~6×10^6^ candidates, each of which will be later compared to each of the training set sequences. This results in a massive load of computation and is likely to exceed the capabilities of the current standard laboratory computer.

To reduce the computational effort and improve the specificity of the search, we implemented a collection of pruning heuristics strategies that are concentrated at two points along the pipeline:

- **Early rejection** criteria are called after the algorithm has identified all subgraph induced CCs and before DTW subsequence matching. The purpose of early rejection is to identify and discard whole sequences or subsequences of candidates that cannot contain the searched motif, and therefore applying DTW subsequence matching to them is pointless.
- **Late rejection** criteria are called after DTW subsequence matching and before 1NN-classification. The purpose of late rejection is to identify and discard subsequences that were matched to the template by the DTW algorithm that cannot be a true appearance of the motif, and therefore they are necessarily false detections and need not participate in the 1NN-classification.

Sequences (or subsequences) that fail to meet all used criteria are immediately labeled as ‘false’ and excluded from the rest of the pipeline, thus significantly reducing computational load. We describe in detail two pruning criteria which provide high reduction of redundant candidates.

***Topological pruning:*** The number of connected graphs of k (unlabeled) vertices, which corresponds to the number of possible topologies of CCs with k cells, increases exponentially with k (see OEIS [A001349](http://oeis.org/A001349) [6]). However, multicellular motifs typically exhibit a repertoire of not more than several topological configurations, thus rendering the vast majority of the search unnecessary. Allowing the user to limit the topological search space by specifying restrictions on valid topologies and skip time points in which candidates do not meet the restrictions can significantly enhance the search. In graph theory terms, this reduces to testing whether the adjacency graph of the candidate is isomorphic to any of the specified valid or invalid ones.

***Temporal overlap pruning:*** By default, DTW based subsequence matching returns all the alignments that are local optima of the search space, which is likely to result in many temporally overlapping candidates for each candidate. This number further exacerbates in multicellular motifs, as these require matching all k! permutations of the template to each candidate. As the motifs carried by the cells are assumed to be temporally discrete (i.e. a group of cells can only perform the same motif one at a time), at any given time point only one subsequence can represent a true motif, at most. This opens the opportunity to discard all other candidates prior to the 1NN classification step and reduce computational load. To do so, our algorithm offers the user the ability to filter the set of candidates from each candidate for the maximal number of non-overlapping candidates having the lowest dissimilarity score, and are therefore the most likely to be true motifs. This was done by implementing a greedy weighted interval scheduler with weights defined as inverse of the dissimilarity score.

***The complete list of pruning heuristics:***

1. *Maximal dissimilarity score threshold:* (late rejection) removes a candidate if its dissimilarity score to the template is above the specified threshold.
2. *Minimal and maximal duration of candidate:* (early and late rejection) removes a candidate if it is shorter / longer than the specified limits.
3. *Temporal overlap pruning:* (late rejection) described above.
4. *Topological pruning:* (early rejection) described above.
5. *Force neighbor exchange:* (early and late rejection) removes a candidate sequence if its topology is constant over its entire existence.
6. *Minimal and maximal number of neighbors per cell:* (early rejection) removes any time point within the lifetime of a candidate sequence in which not all cells meet the specified range. Note that this refers to neighbors that are also cells within the inspected candidate.

For the search of T1-transitions and T1-reversals we used the following pruning mechanisms: topological pruning, force neighbor exchange, minimal duration of candidate: 2.5 minutes, temporal overlap pruning.

For the search of 5- and 6-cell rosettes we used the following pruning mechanisms: force neighbor exchange, minimal duration of candidate: 2.5 minutes, temporal overlap pruning, minimal number of neighbors per cell: 2.

**Rule based candidate filtering**

An additional option for accuracy refinement is by implementing rule-based filters on the time-series values of the candidates. Specifically, for T1-transitions and T1-reversals we require that the length of both contracting and growing junctions will reach at least 2 microns, and for 5- and 6-cell rosettes we require that the maximal cell boundary distance at the point of transition will be 2- and 3-microns at most, respectively.

**ALGORITHM OUTPUT:**

For each identified appearance of the motif (i.e. a candidate that was labeled *true* during the 1NN classification), the algorithm provides the following list of items:

1. The indices of the cells composing the candidate. The order of the indices reflects the optimal matching between the cells of the candidate and the cells of the template.
2. The timeframe (i.e. indices of first and last movie frames) during which the motif was manifested.
3. The time-point matching (i.e. the warping path) between the candidate and the template.
4. The values of all extracted features throughout the progression of the motif.

***Automated propagation of temporal labeling:*** A key advantage of the DTW algorithm is the time-point matching (i.e. the warping path) between the template and the identified subsequence. This is particularly useful when the downstream analysis of identified appearances requires the identification of specific time points throughout its progression. For instance, to calculate the dynamics of junction contraction and elongation in T1-transitions (fig. 4B,E and 5B) it is first necessary to identify the exact time point of transition (i.e. when neighbor exchange occurs). As demonstrated in figures 1B,C and figures 3A,B, manual identification of the point of transition within the sequence of the template can be automatically propagated to each identified appearance of the motif based on the warping path. To evaluate the accuracy of this temporal propagation we calculated the time difference between the time of transition as indicated by our algorithm and manual control:


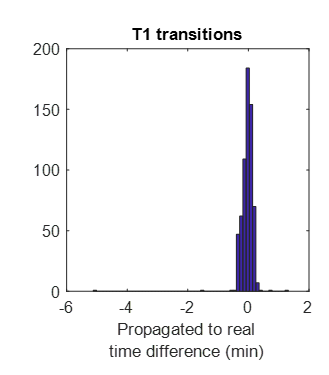


As evident from the histogram, the average error in time propagation (mean ± SD = 0.46 ± 9.36 seconds) is very low and therefore reliable.

**BENCHMARK ANALYSIS USING VERTEX MODEL SIMULATION**

To test the performance of our algorithm we applied it on a vertex-model simulated tissue. To this end We used the model described in [7] with the following parameters: velocity of cell self propulsion (v0) = 0.5, rotational diffusion constant (Dr) = 0.1, preferred cell shape parameter (p0) = 3.82 and cell area modulus (KA) = 100. The tissue included 1006 cells confined within a circular boundary that performed a total of 501 T1-transitions over 100 frames. Our algorithm has identified 488 true events, resulting in a true positive rate of 97.4%. Manual examination of the remaining 13 (2.6%) events showed that in all cases either the initial length of the contracting junction or the final length reached by the newly formed junction were approximately 1-2 pixels, and thus below the sensitivity threshold of our image segmentation module. Notably, no false detections of T1-transitions were made. To evaluate our algorithm’s accuracy in time-point matching, we compared the time point of transitions in each identified T1 as predicted by our algorithm to that reported by the simulation algorithm. In 15/488 (3.1%) an error of 1 frame had occurred, while in the remaining 473/488 (96.9%) no error was made. This demonstrates the high specificity and sensitivity of our algorithm in detecting multicellular motifs, as well as its temporal accuracy in automatic timepoint labeling of events throughout the progression of the identified motifs.

**LITERATURE**

1. Sommer C, Straehle C, Kothe U, Hamprecht FA. Ilastik: Interactive learning and segmentation toolkit. Proc - Int Symp Biomed Imaging. 2011; 230–233. doi:10.1109/ISBI.2011.5872394

2. Thirion JP. Image matching as a diffusion process: An analogy with Maxwell’s demons. Med Image Anal. 1998;2: 243–260. doi:10.1016/S1361-8415(98)80022-4

3. Keller F, Müller E, Wixler A, Böhm K. Flexible and adaptive subspace search for outlier analysis. Int Conf Inf Knowl Manag Proc. 2013. doi:10.1145/2505515.2505560

4. Karakashia S. An Implementation of An Algorithm for Generating All Connected Subgraphs of a Fixed Size. 2010. Available: http://consystlab.unl.edu/our_work/software.html

5. Niennattrakul V, Wanichsan D, Ratanamahatana CA. Accurate subsequence matching on data stream under time warping distance. Lecture Notes in Computer Science (including subseries Lecture Notes in Artificial Intelligence and Lecture Notes in Bioinformatics). 2010. pp. 156–167. doi:10.1007/978-3-642-14640-4_12

6. Sloane NJA. The encyclopedia of integer sequences. In: Choice Reviews Online [Internet]. 1995 [cited 26 Oct 2018] pp. 33-2176-33–2176. doi:10.5860/choice.33-2176

7. Bi D, Yang X, Marchetti MC, Manning ML. Motility-driven glass and jamming transitions in biological tissues. Phys Rev X. 2016;6. doi:10.1103/PhysRevX.6.021011
