# Supplementary material for: Template-based mapping of dynamic motifs in tissue morphogenesis
Source: PLoS Comput Biol. 2020 Aug 21;16(8):e1008049. doi: 10.1371/journal.pcbi.1008049 (PMC7442231; doi:10.1371/journal.pcbi.1008049)
Supplement: S2 File — (PPTX) [file pcbi.1008049.s002.pptx]

## Slide 1
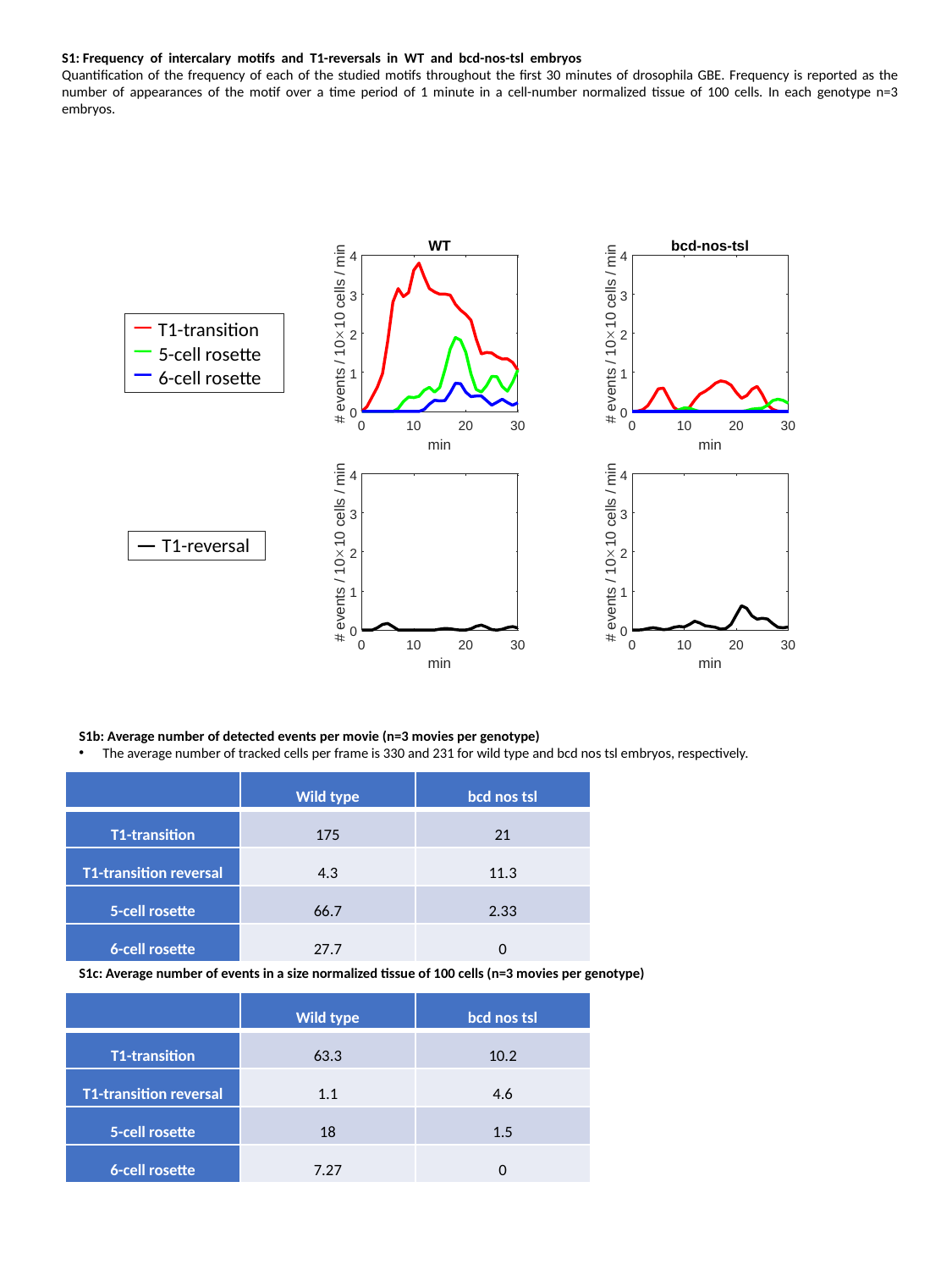

S1: Frequency of intercalary motifs and T1-reversals in WT and bcd-nos-tsl embryos
Quantification of the frequency of each of the studied motifs throughout the first 30 minutes of drosophila GBE. Frequency is reported as the number of appearances of the motif over a time period of 1 minute in a cell-number normalized tissue of 100 cells. In each genotype n=3 embryos.
T1-transition
5-cell rosette
6-cell rosette
T1-reversal
S1b: Average number of detected events per movie (n=3 movies per genotype)
The average number of tracked cells per frame is 330 and 231 for wild type and bcd nos tsl embryos, respectively.
S1c: Average number of events in a size normalized tissue of 100 cells (n=3 movies per genotype)
| | Wild type | bcd nos tsl |
| --- | --- | --- |
| T1-transition | 175 | 21 |
| T1-transition reversal | 4.3 | 11.3 |
| 5-cell rosette | 66.7 | 2.33 |
| 6-cell rosette | 27.7 | 0 |
| | Wild type | bcd nos tsl |
| --- | --- | --- |
| T1-transition | 63.3 | 10.2 |
| T1-transition reversal | 1.1 | 4.6 |
| 5-cell rosette | 18 | 1.5 |
| 6-cell rosette | 7.27 | 0 |

## Slide 2
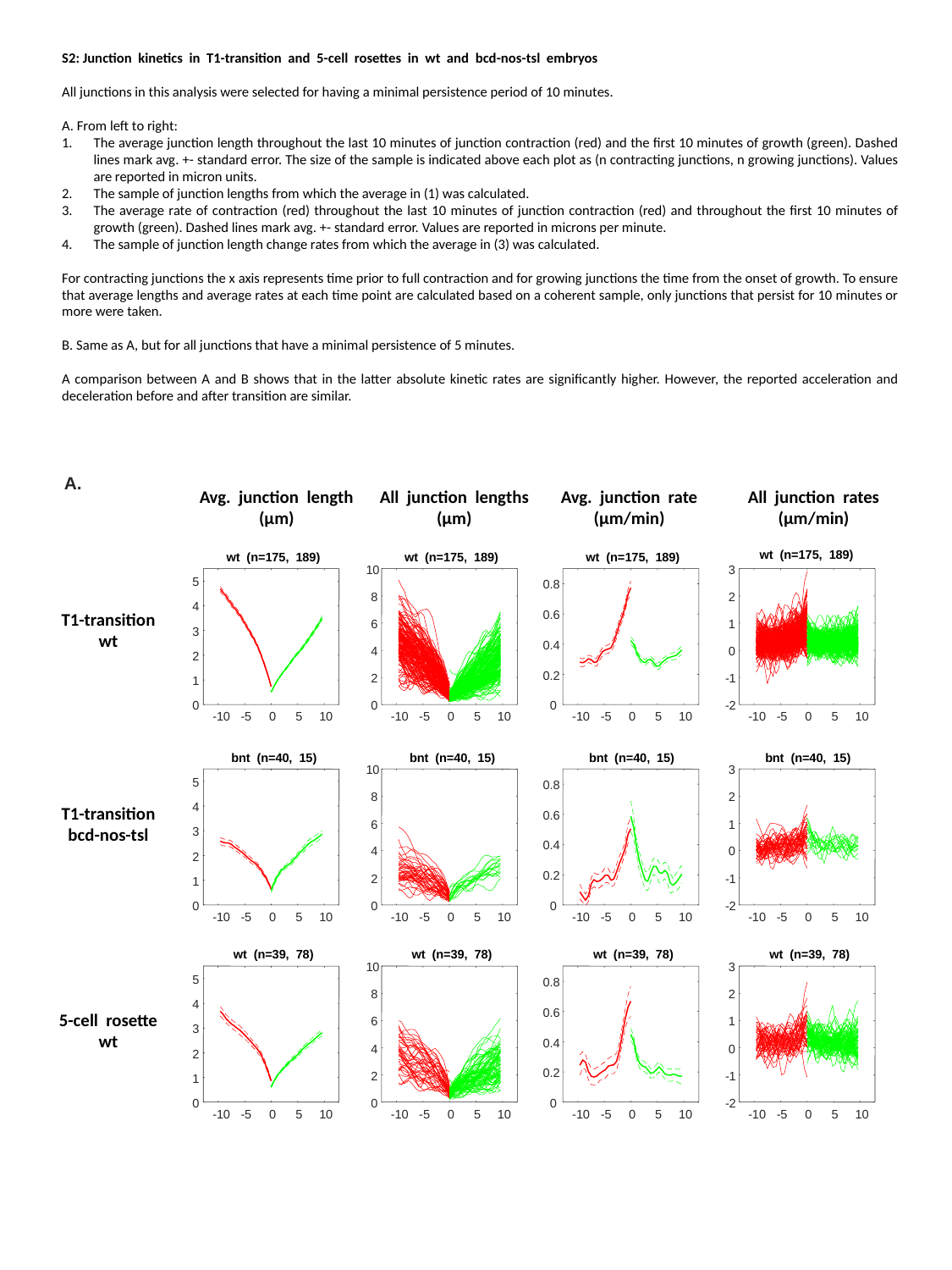

S2: Junction kinetics in T1-transition and 5-cell rosettes in wt and bcd-nos-tsl embryos
All junctions in this analysis were selected for having a minimal persistence period of 10 minutes.
A. From left to right:
The average junction length throughout the last 10 minutes of junction contraction (red) and the first 10 minutes of growth (green). Dashed lines mark avg. +- standard error. The size of the sample is indicated above each plot as (n contracting junctions, n growing junctions). Values are reported in micron units.
The sample of junction lengths from which the average in (1) was calculated.
The average rate of contraction (red) throughout the last 10 minutes of junction contraction (red) and throughout the first 10 minutes of growth (green). Dashed lines mark avg. +- standard error. Values are reported in microns per minute.
The sample of junction length change rates from which the average in (3) was calculated.
For contracting junctions the x axis represents time prior to full contraction and for growing junctions the time from the onset of growth. To ensure that average lengths and average rates at each time point are calculated based on a coherent sample, only junctions that persist for 10 minutes or more were taken.
B. Same as A, but for all junctions that have a minimal persistence of 5 minutes.
A comparison between A and B shows that in the latter absolute kinetic rates are significantly higher. However, the reported acceleration and deceleration before and after transition are similar.
A.
Avg. junction length
(µm)
All junction lengths
(µm)
Avg. junction rate
(µm/min)
All junction rates
(µm/min)
wt (n=175, 189)
wt (n=175, 189)
wt (n=175, 189)
wt (n=175, 189)
10
3
5
0.8
8
2
4
T1-transition
wt
0.6
6
1
3
0.4
4
0
2
0.2
2
-1
1
0
0
0
-2
-10
-5
0
5
10
-10
-5
0
5
10
-10
-5
0
5
10
-10
-5
0
5
10
bnt (n=40, 15)
bnt (n=40, 15)
bnt (n=40, 15)
bnt (n=40, 15)
10
3
5
0.8
8
2
T1-transition
bcd-nos-tsl
4
0.6
6
1
3
0.4
4
0
2
0.2
2
-1
1
0
0
0
-2
-10
-5
0
5
10
-10
-5
0
5
10
-10
-5
0
5
10
-10
-5
0
5
10
wt (n=39, 78)
wt (n=39, 78)
wt (n=39, 78)
wt (n=39, 78)
10
3
5
0.8
8
2
4
5-cell rosette
wt
0.6
6
1
3
0.4
4
0
2
0.2
2
-1
1
0
0
0
-2
-10
-5
0
5
10
-10
-5
0
5
10
-10
-5
0
5
10
-10
-5
0
5
10

## Slide 3
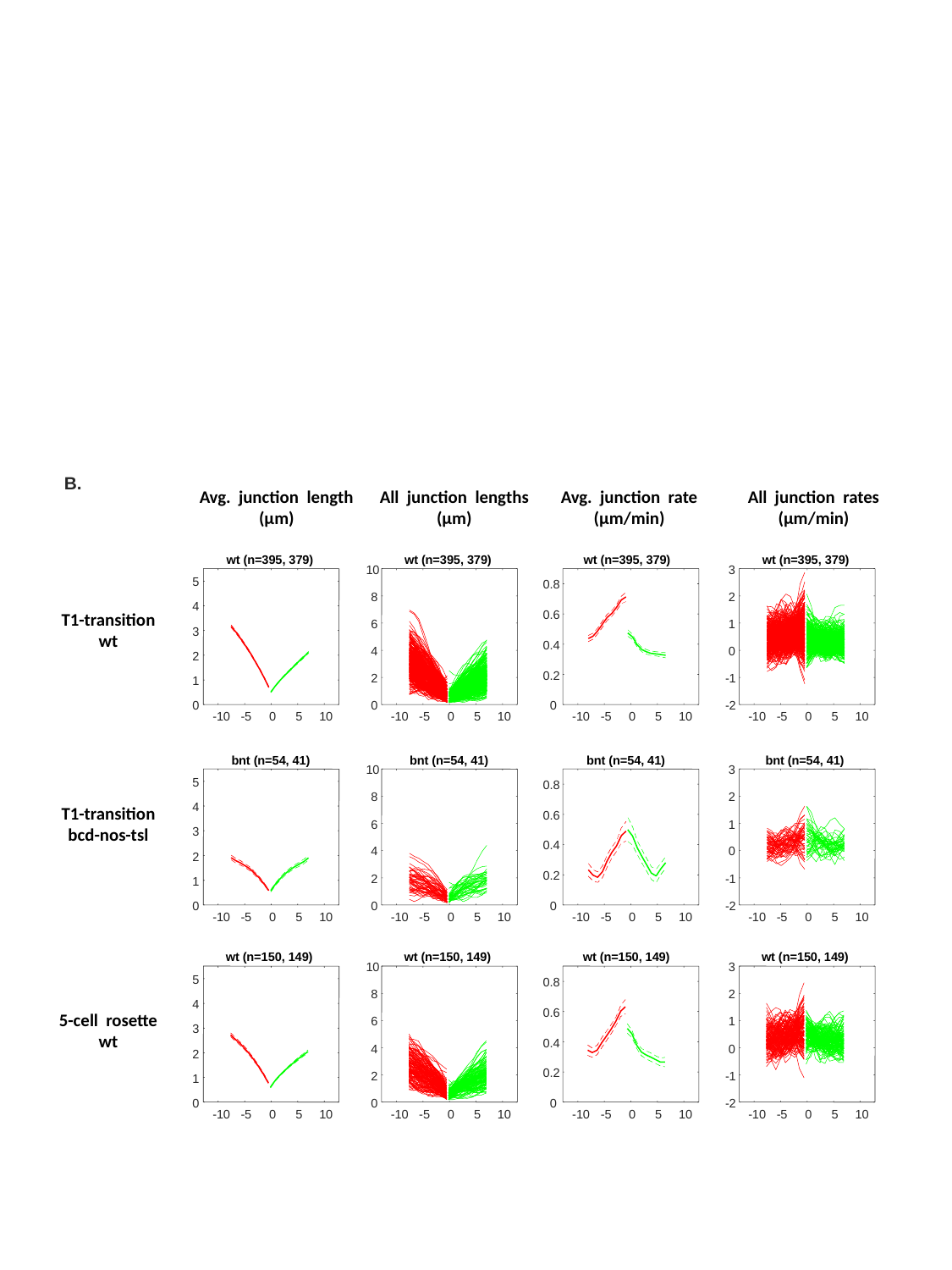

B.
Avg. junction length
(µm)
All junction lengths
(µm)
Avg. junction rate
(µm/min)
All junction rates
(µm/min)
wt (n=395, 379)
wt (n=395, 379)
wt (n=395, 379)
wt (n=395, 379)
10
3
5
0.8
8
2
4
T1-transition
wt
0.6
6
1
3
0.4
4
0
2
0.2
2
-1
1
0
0
0
-2
-10
-5
0
5
10
-10
-5
0
5
10
-10
-5
0
5
10
-10
-5
0
5
10
bnt (n=54, 41)
bnt (n=54, 41)
bnt (n=54, 41)
bnt (n=54, 41)
10
3
5
0.8
8
2
T1-transition
bcd-nos-tsl
4
0.6
6
1
3
0.4
4
0
2
0.2
2
-1
1
0
0
0
-2
-10
-5
0
5
10
-10
-5
0
5
10
-10
-5
0
5
10
-10
-5
0
5
10
wt (n=150, 149)
wt (n=150, 149)
wt (n=150, 149)
wt (n=150, 149)
10
3
5
0.8
8
2
4
5-cell rosette
wt
0.6
6
1
3
0.4
4
0
2
0.2
2
-1
1
0
0
0
-2
-10
-5
0
5
10
-10
-5
0
5
10
-10
-5
0
5
10
-10
-5
0
5
10

## Slide 4
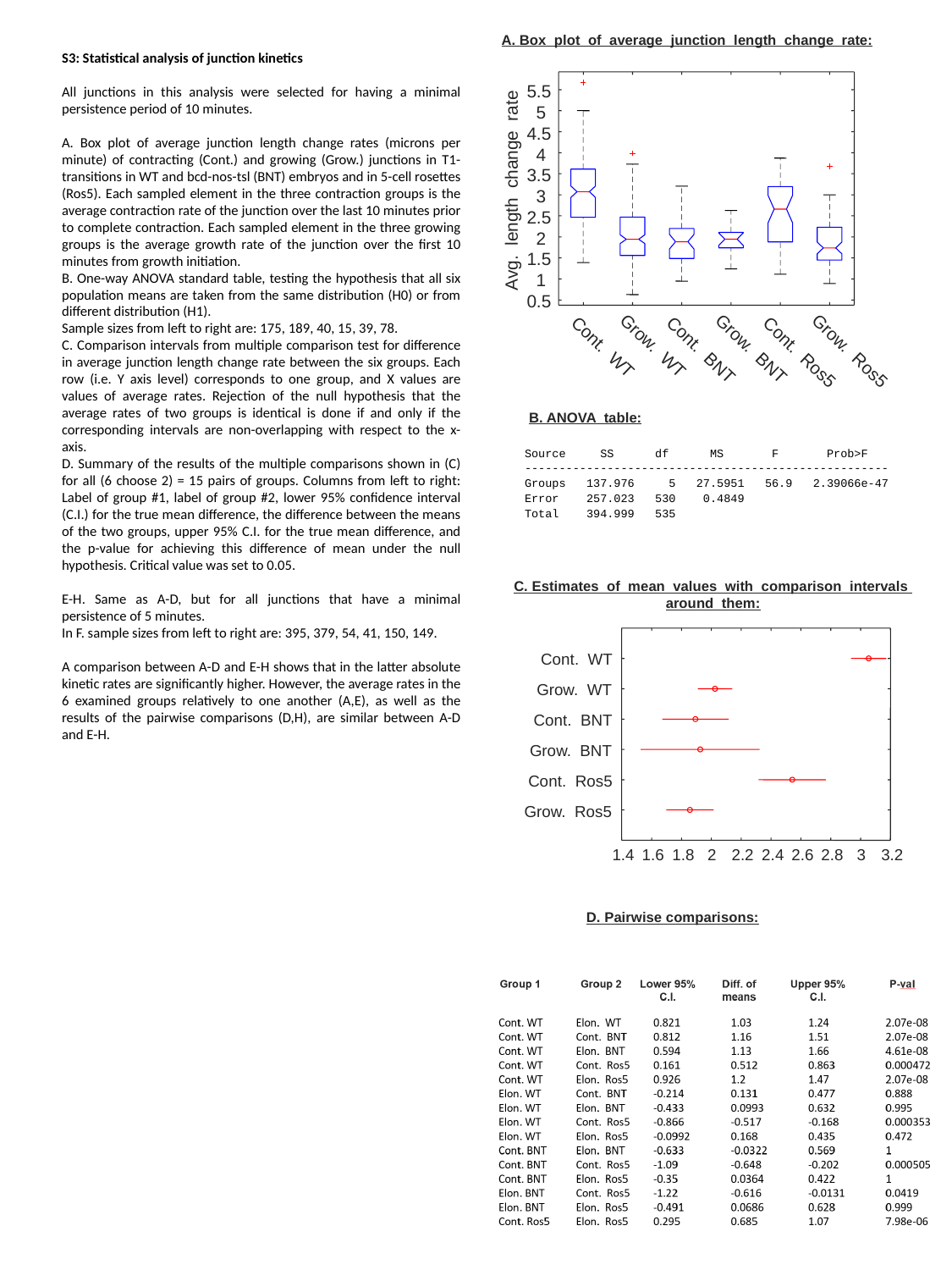

A. Box plot of average junction length change rate:
S3: Statistical analysis of junction kinetics
All junctions in this analysis were selected for having a minimal persistence period of 10 minutes.
A. Box plot of average junction length change rates (microns per minute) of contracting (Cont.) and growing (Grow.) junctions in T1-transitions in WT and bcd-nos-tsl (BNT) embryos and in 5-cell rosettes (Ros5). Each sampled element in the three contraction groups is the average contraction rate of the junction over the last 10 minutes prior to complete contraction. Each sampled element in the three growing groups is the average growth rate of the junction over the first 10 minutes from growth initiation.
B. One-way ANOVA standard table, testing the hypothesis that all six population means are taken from the same distribution (H0) or from different distribution (H1).
Sample sizes from left to right are: 175, 189, 40, 15, 39, 78.
C. Comparison intervals from multiple comparison test for difference in average junction length change rate between the six groups. Each row (i.e. Y axis level) corresponds to one group, and X values are values of average rates. Rejection of the null hypothesis that the average rates of two groups is identical is done if and only if the corresponding intervals are non-overlapping with respect to the x-axis.
D. Summary of the results of the multiple comparisons shown in (C) for all (6 choose 2) = 15 pairs of groups. Columns from left to right: Label of group #1, label of group #2, lower 95% confidence interval (C.I.) for the true mean difference, the difference between the means of the two groups, upper 95% C.I. for the true mean difference, and the p-value for achieving this difference of mean under the null hypothesis. Critical value was set to 0.05.
E-H. Same as A-D, but for all junctions that have a minimal persistence of 5 minutes.
In F. sample sizes from left to right are: 395, 379, 54, 41, 150, 149.
A comparison between A-D and E-H shows that in the latter absolute kinetic rates are significantly higher. However, the average rates in the 6 examined groups relatively to one another (A,E), as well as the results of the pairwise comparisons (D,H), are similar between A-D and E-H.
5.5
5
4.5
4
3.5
Avg. length change rate
3
2.5
2
1.5
1
0.5
Grow. WT
Cont. WT
Grow. BNT
Cont. BNT
Grow. Ros5
Cont. Ros5
B. ANOVA table:
C. Estimates of mean values with comparison intervals around them:
Cont. WT
Grow. WT
Cont. BNT
Grow. BNT
Cont. Ros5
Grow. Ros5
1.4
1.6
1.8
2
2.2
2.4
2.6
2.8
3
3.2
D. Pairwise comparisons:

## Slide 5
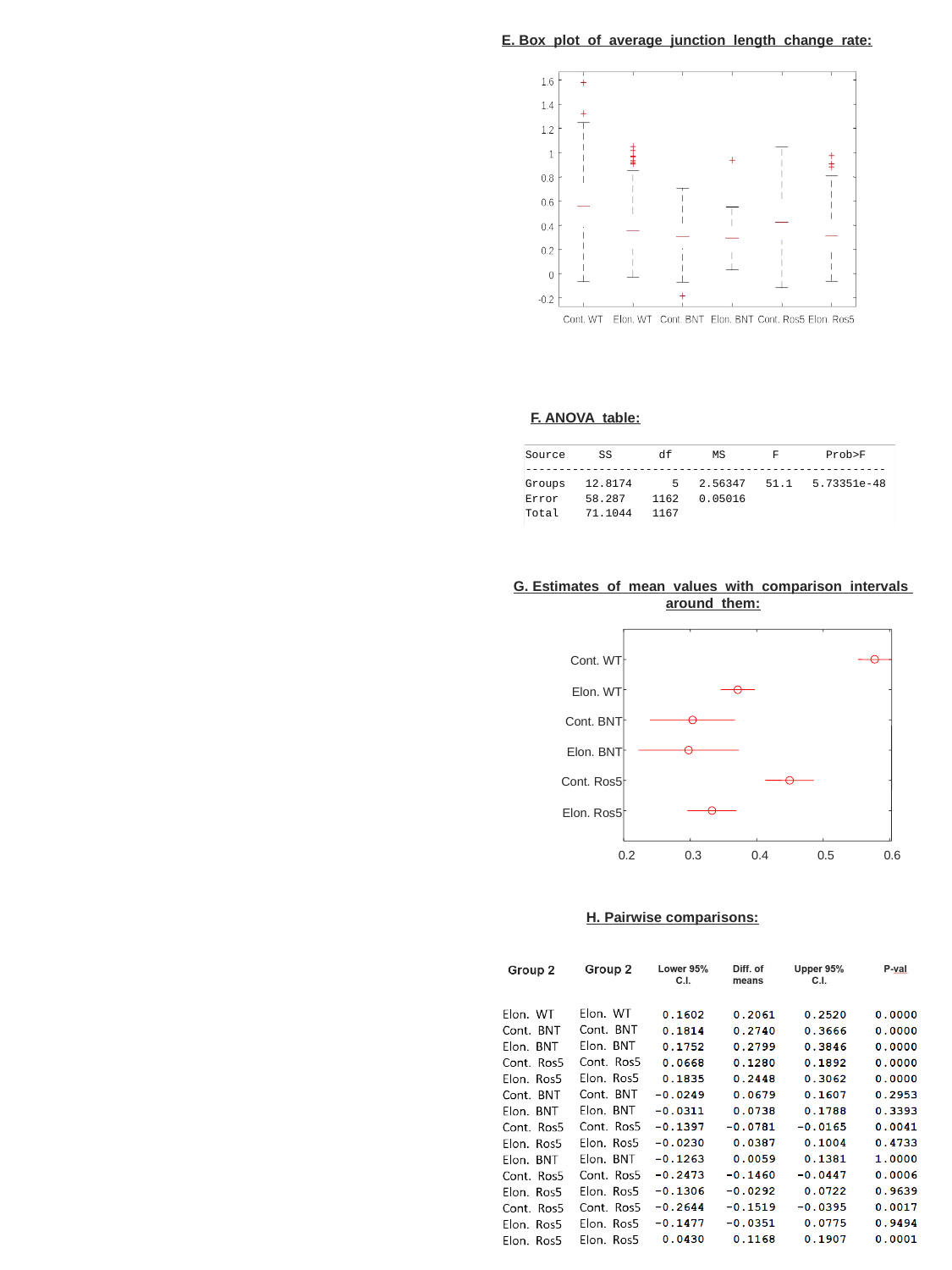

E. Box plot of average junction length change rate:
F. ANOVA table:
G. Estimates of mean values with comparison intervals around them:
Cont. WT
Elon. WT
Cont. BNT
Elon. BNT
Cont. Ros5
Elon. Ros5
0.2
0.3
0.4
0.5
0.6
H. Pairwise comparisons:

## Slide 6
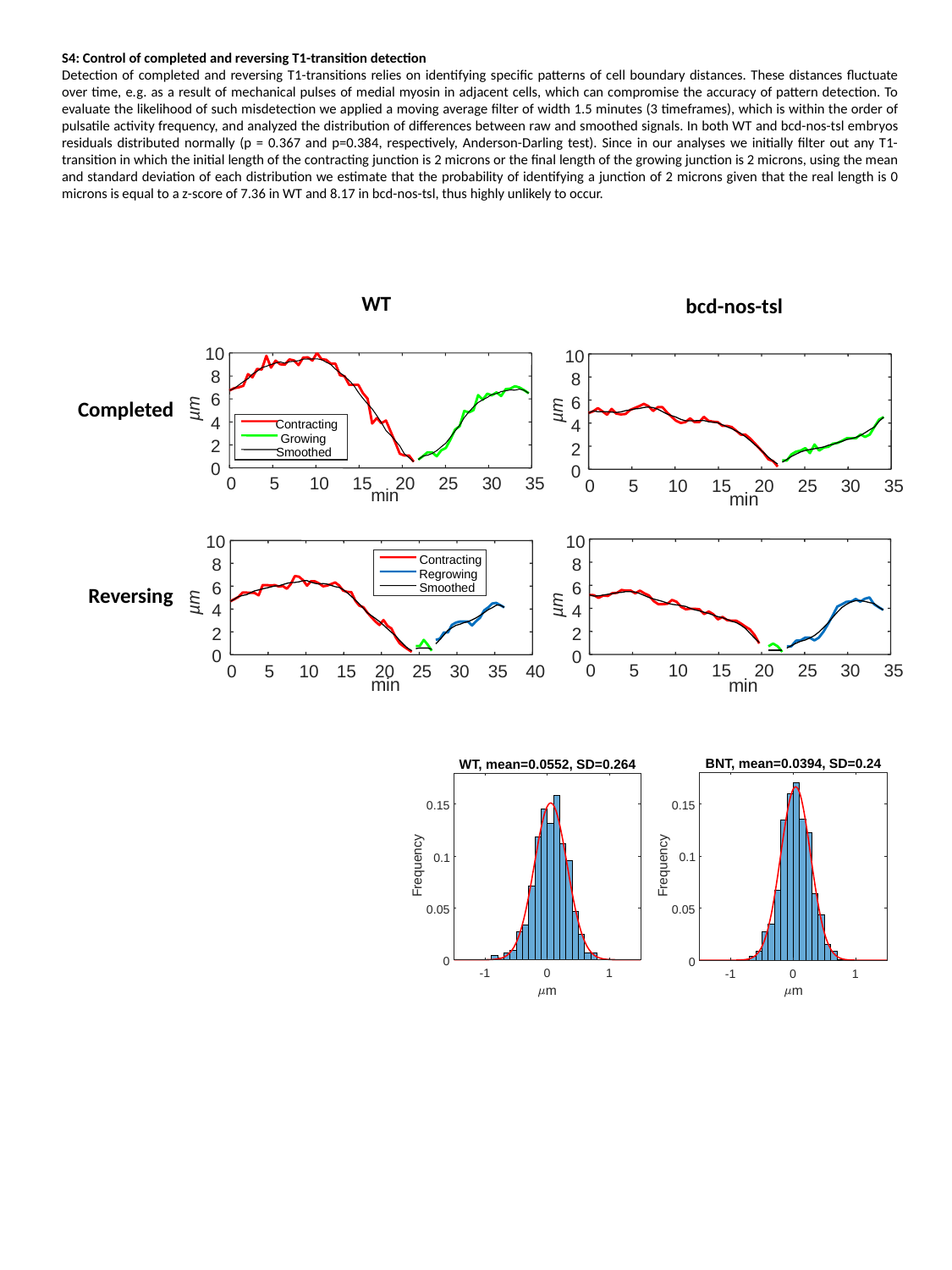

S4: Control of completed and reversing T1-transition detection
Detection of completed and reversing T1-transitions relies on identifying specific patterns of cell boundary distances. These distances fluctuate over time, e.g. as a result of mechanical pulses of medial myosin in adjacent cells, which can compromise the accuracy of pattern detection. To evaluate the likelihood of such misdetection we applied a moving average filter of width 1.5 minutes (3 timeframes), which is within the order of pulsatile activity frequency, and analyzed the distribution of differences between raw and smoothed signals. In both WT and bcd-nos-tsl embryos residuals distributed normally (p = 0.367 and p=0.384, respectively, Anderson-Darling test). Since in our analyses we initially filter out any T1-transition in which the initial length of the contracting junction is 2 microns or the final length of the growing junction is 2 microns, using the mean and standard deviation of each distribution we estimate that the probability of identifying a junction of 2 microns given that the real length is 0 microns is equal to a z-score of 7.36 in WT and 8.17 in bcd-nos-tsl, thus highly unlikely to occur.
WT
bcd-nos-tsl
10
10
8
8
6
Completed
6
µm
µm
4
4
Contracting
Growing
2
2
Smoothed
0
0
0
5
10
15
20
25
30
35
0
5
10
15
20
25
30
35
min
min
10
10
Contracting
8
8
Regrowing
Reversing
6
6
Smoothed
µm
µm
4
4
2
2
0
0
0
5
10
15
20
25
30
35
0
5
10
15
20
25
30
35
40
min
min
