# Supplementary material for: Template-based mapping of dynamic motifs in tissue morphogenesis
Source: PLoS Comput Biol. 2020 Aug 21;16(8):e1008049. doi: 10.1371/journal.pcbi.1008049 (PMC7442231; doi:10.1371/journal.pcbi.1008049)
Supplement: S3 File — (DOCX) [file pcbi.1008049.s003.docx]

**SUPPLEMENTARY FILE 3:**

**Automated mapping and phase labeling of dividing nuclei and ingressing neuroblasts**

Since our mapping strategy relies on template matching rather than detection of topological changes, it can also be used to map complex temporal patterns that do not have simple topological signatures. To demonstrate this capacity, we analyzed cell cycle progression during nuclear cleavage cycles 11-13, using histone labeled embryos. As nuclear geometry and histone distribution patterns differ between cycles, we selected a representative nucleus from each cycle to serve as template [1] and matched it to other nuclei in the video using cell area, and mean fluorescence intensity. Our algorithm identified a total of 81, 157 and 281 nuclei in cycles 11-13, respectively. Based on the time point matching indicated by the warping path of each match, we could automatically label each nucleus with respect to its phase in the cell cycle. Our analysis confirms the previously described elongation of interphase periods from cycle 11-13 (avg.±S.D = 5.4±0.3, 7.9±0.6, 13.6±0.8 min, respectively), while the durations of mitoses remain unchanged (avg.±S.D = 4.2±0.3 4.1±0.6 4.2±1.0 min, respectively) in agreement with [2]). Taken together, our analyses highlight the broad applicability of our template matching algorithm to studying the distribution and the dynamics of the basic behaviors underlying tissue development.

**Figure caption:** Mapping dividing nuclei during cleavage cycle 12 (live image is courtesy of Nareg Djabrayan). Top: the time series of the template. Apical cell area (brown) and mean fluorescence intensity (blue) were used as features. Bottom: multiple sequence alignments of the top three matches. Left arrow indicates that the sequence had to be temporally stretched by repeating the previous time point. Each column represents the same biological phase in all sequences, thus allowing to automatically and accurately propagate time point labels from the template to each identified match.


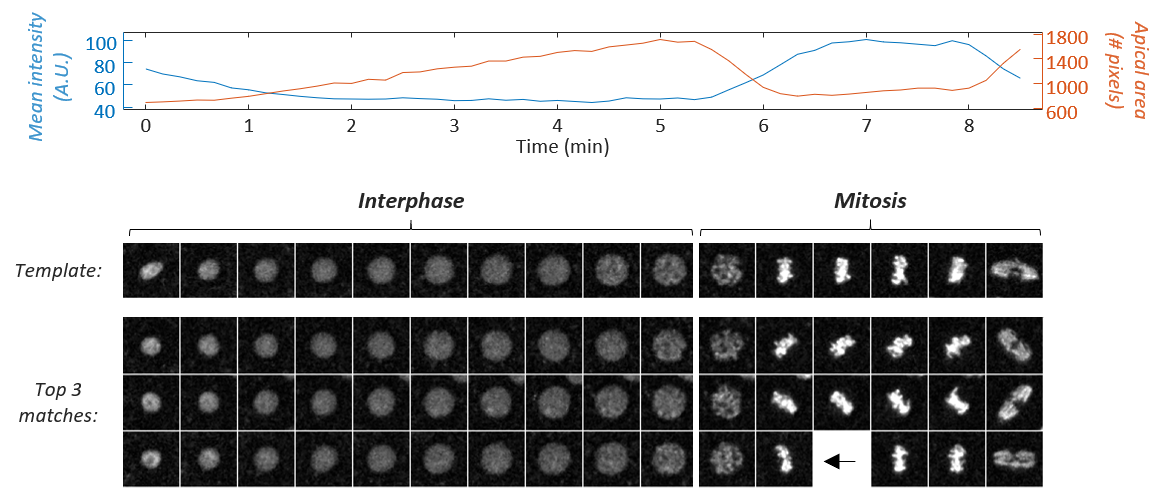


To map ingressing neuroblasts in membrane labeled embryos during late GBE we selected cell area as the sole feature. The area curve of individual neuroblasts is typically noisy and irregular, and therefore a template based on a real cell may not perform well. Instead, we drew the feature manually as a straight decreasing line, while imitating the degree of the slope and the duration we observed in the live data. Multiple sequence alignment of the top matches in both motifs show excellent temporal match, which can then be used to automatically and accurately propagate time point labels from the template to each identified instance.


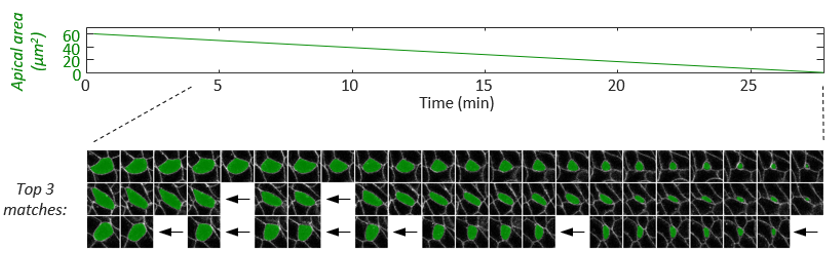
**Figure caption:** A demonstration of mapping ingressing neuroblasts during late GBE. Top: the time series of the template. Bottom: multiple sequence alignment of the top three matches. Left arrows indicate that the sequence had to be temporally stretched by repeating the previous time point, which allows to infer the relative dynamics of the sequences.

**LITERATURE**

1. Shindo Y, Amodeo AA. Dynamics of Free and Chromatin-Bound Histone H3 during Early Embryogenesis. Curr Biol. 2019;29: 359-366.e4. doi:10.1016/j.cub.2018.12.020

2. Djabrayan NJV, Smits CM, Krajnc M, Stern T, Yamada S, Lemon WC, et al. Metabolic Regulation of Developmental Cell Cycles and Zygotic Transcription. Curr Biol. 2019;29: 1193-1198.e5. doi:10.1016/j.cub.2019.02.028
